# Lipid metabolism dysfunction induced by age-dependent DNA methylation accelerates aging

**DOI:** 10.1038/s41392-022-00964-6

**Published:** 2022-05-25

**Authors:** Xin Li, Jiaqiang Wang, LeYun Wang, Yuanxu Gao, Guihai Feng, Gen Li, Jun Zou, Meixin Yu, Yu Fei Li, Chao Liu, Xue Wei Yuan, Ling Zhao, Hong Ouyang, Jian-Kang Zhu, Wei Li, Qi Zhou, Kang Zhang

**Affiliations:** 1 State Key Laboratory of Stem Cell and Reproductive Biology, Institute of Zoology, Chinese Academy of Sciences, 100101 Beijing, China; 2 Institute for Genomic Medicine, University of California San Diego, La Jolla, CA 92037 USA; 3 Faculty of Medicine, Macau University of Science and Technology, Tapai, Macau, 999078 China; 4 State Key Laboratory of Lunar and Planetary Sciences, Macau University of Science and Technology, Tapai, Macau, 999078 China; 5 Clinical Translational Innovation Center, West China Hospital, Sichuan University, Chengdu, 610041 China; 6 Guangzhou Women and Children Medical Center, Guangzhou Medical University, Guangzhou, 510623 China; 7 Zhongshan Ophthalmic Center, Sun Yat-sen University, Guangzhou, 510060 China; 8 Institute of Advanced Biotechnology and School of Life Sciences, Southern University of Science and Technology, Shenzhen, 518055 China

**Keywords:** Ageing, Epigenetics, Ageing, Bioinformatics

## Abstract

Epigenetic alterations and metabolic dysfunction are two hallmarks of aging. However, the mechanism of how their interaction regulates aging, particularly in mammals, remains largely unknown. Here we show ELOVL fatty acid elongase 2 (Elovl2), a gene whose epigenetic alterations are most highly correlated with age prediction, contributes to aging by regulating lipid metabolism. We applied artificial intelligence to predict the protein structure of ELOVL2 and the interaction with its substrate. Impaired Elovl2 function disturbs lipid synthesis with increased endoplasmic reticulum stress and mitochondrial dysfunction, leading to key aging phenotypes at both cellular and physiological level. Furthermore, restoration of mitochondrial activity can rescue age-related macular degeneration (AMD) phenotypes induced by Elovl2 deficiency in human retinal pigmental epithelial (RPE) cells; this indicates a conservative mechanism in both human and mouse. Taken together, we revealed an epigenetic-metabolism axis contributing to aging and illustrate the power of an AI-based approach in structure-function studies.

## Introduction

Aging is an inevitable life process characterized by increasing vulnerability to disease, loss of molecular fidelity, and progressive decline in tissue and organ function.^1^ Epigenetic alterations play a key role in aging by integrating environmental signals to regulate gene expression and downstream cellular processes.^2–6^ The integral relationship between aging and DNA methylation levels was not clearly described until recently,^7–10^ Several hundred CpG sites with DNA methylation levels correlating to biological age were precisely mapped.^6,11^ Studies by several groups established epigenetic DNA methylation signatures^12^ that can serve as an accurate biological age “clock” in many different tissue types,^13^ see recent review by Bell et al.^14^ some of which were found within metabolism-associated genes which indicates an intimate association between epigenetic alterations and metabolism in aging.

Elovl2, a gene which functions as a master control of poly unsaturated fatty acid (PUFA) synthesis and is strongly associated with diabetes, shows the most relevance to aging.^4,11,15,16^ The DNA methylation status of Elovl2 explains 70% of the “aging epigenetic clock”.^15^ Several CG markers including Elovl2 predict aging in various tissues^15,17^ and are referred to as universal bioage markers,^15,17^ whereas the rest markers are usually tissue specific and carry less weight in aging prediction. Functionally, Elovl2 plays an irreplaceable role in the synthesis of PUFAs, which are critical for a range of biological processes. It has been reported that the concentration of PUFAs in the body correlates negatively with age in human, including both their side chains and by-products.^18,19^ Although the role of Elovl2 in the synthesis of PUFAs has been well documented in previous studies, the effect of Elovl2 deficiency on aging or the downstream mechanism underlying how age-related Elovl2 DNA methylation contributes to aging remains unknown.

Currently, there is lack of protein structural information of ELOVL2 and other ELOVL family members. This limits our understanding of the downstream mechanism of ELOVL2 physiolocal function. Recent exciting achievements in AI-based 3D protein structure predictions have ushed a new era for biology and medicine learning, enabling rapid protein structure predictions and offering tremendous potential for functional studies.^20,21^ We applied this approach to predict and understand ELOVL2 and its interaction with substrates and gain insight into its functions.

We further reasoned that decrease of Elovl2 expression contributed to aging by disrupting the balance of lipid metabolism in ER and mitochondria. On one hand, Elvol2 is localized in the endoplasmic reticulum (ER), where PUFAs are elongated. On the other hand, mitochondria are the main site of lipid degradation where fatty acid oxidation occurs. Both of them play important roles in lipid metabolism and aging. Here, we show that lack of Elovl2 leads to a decline in PUFA synthesis and the accumulation of short fatty acids including PUFA precursors in ER, altering mitochondrial energy metabolism and resulting in chronic ER stress and mitochondria dysfunction. These changes contributed to aging phenotypes including stem cell exhaustion, cognitive decline, retinal degeneration, and glucose intolerance. We have also applied this mechanism in a human RPE cell model and observed a rescue phenotype. In this paper, we revealed an epigenetic-metabolism axis contributing to aging and illustrate the power of an AI-based approach in structure-function studies.

## Results

### Increased DNA methylation with concomitant decreased gene expression of Elovl2 correlates with biological aging

**Fig. 1:**
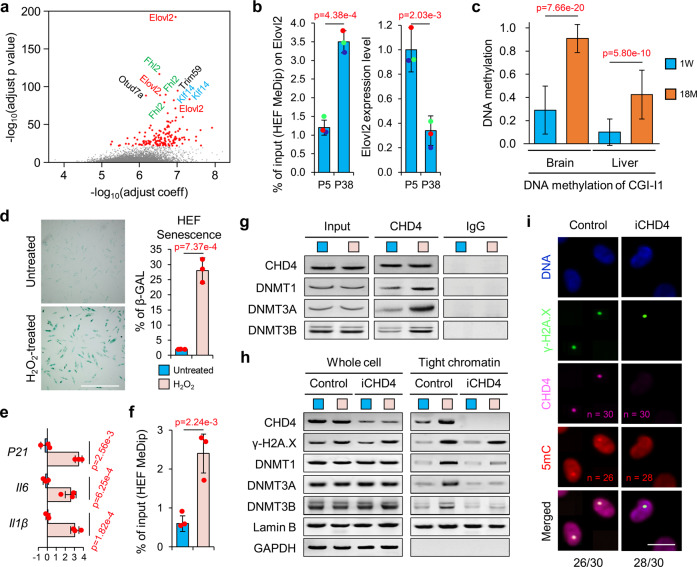
Elovl2 is a metabolic gene that serves as marker of aging. **a** Correlation between DNA methylation of genes and aging. The significant sites are marked. The aging model were described in our previous work.^11^ *p* values are based on a least-squares model built with the same terms and drop-one *F* tests. **b** MeDip-qPCR and qPCR of Elovl2 in human fibroblasts (*n* = 3). Error bars, standard error of the mean (SEM). Levels of significance were calculated with one-tailed Student’s *t* test. The expression and DNA methylation based on the same plates of cells, which are inked with the same color. **c** DNA methylation level on the CpG island in intron 1 (CGI-I1) of Elovl2 in the brain and liver of 129/sv mice. For each group 30 Sanger sequencing results from six mice were used. Error bars, SEM. Levels of significance were calculated with two-tailed Student’s *t* test. **d** Beta-galactosidase (β-GAL) staining on young (p5) human fibroblasts with or without hydrogen peroxide (H_2_O_2_) treatment (*n* = 3). Scale bar, 100 µm. Levels of significance were calculated with one-tailed Student’s *t* test. **e** qPCR showing the transcriptional changes of cellular senescence markers in normal and H_2_O_2_ treated human fibroblasts (*n* = 3). Error bars, SEM. Levels of significance were calculated with two-tailed Student’s *t* test. **f** MeDip-qPCR of Elovl2 in H_2_O_2_ treated human fibroblasts (*n* = 3). Error bars. Levels of significance were calculated with one-tailed Student’s *t* test. **g** Co-immunoprecipitation on human fibroblasts with or without H_2_O_2_ treatment. **h** Western blotting showing that the H_2_O_2_-induced accumulation of DNMTs to the chromatins was CHD4-dependent. **i** The recruitment of CHD4 and 5mC on DNA damage sites of cells irradiated with a 450 nm laser. Scale bar, 5 µm. For each group, 30 cells were treated. 26 out of 30 cells in control group show co-localization of CDH4 and 5mC to γ-H2A.X site, while 28 out of 30 cells in iCDH4 group have no accumulation of CDH4 and 5mC to γ-H2A.X site

We and others have reported a group of genes in human and mice for which DNA methylation level corelated with physiological aging status; consequently, these genes can serve as predictors for biological age.^6,11^ Consistent with previous reports,^4,11,15^ we observed that, lipid storage, fatty acid metabolism and lipogenesis associated genes were revealed in Gene Ontology (GO) term analysis of top relevant predictor genes. We analyzed the gene list of the top predictors, among the top predictor genes, the Elovl2 locus showed the most significant relevance with biological age (Fig. 1a and Supplementary Fig. 1a). In order to study the mechanistic connection between age-related genes and aging process we established both human and mouse models. A similar age-related DNA methylation pattern was confirmed in the human in vitro cell model and the mouse in-vivo model. By using a human fibroblast cell model, a dramatic increase of DNA methylation on Elovl2 accompanied with downregulated Elovl2 expression level was detected in aged human fibroblasts (38 passages) (Fig. 1b). Next, we examined CpG islands within the Elovl2 gene in different mouse tissues at different age: CGI-I1 in the first intron, and CGI-E3, E4, and E8 in the 3rd, 4th and 8th exons (Supplementary Fig. 1b). We found that the DNA methylation of CGI-I1, E3, E4, and E8 in brain and liver significantly increased with aging in 129/sv strains (Fig. 1c and Supplementary Fig. 1c, d). Besides, qPCR analysis showed that the expression level of Elovl2 decreased with aging in 129/sv mice (Supplementary Fig. 1e). A consistent result was observed from ICR mouse strain (Supplementary Fig. 1c–e). These results indicated that Elovl2 loci showed a conserved pattern of increased DNA methylation and reduced expression in both the human fibroblast cell model and the mouse model.

### DNA methylation of Elovl2 is mediated during cellular senescence and the DNA damage repair process

Next, we investigated how environmental factors during the aging process affect DNA methylation of Elovl2. It has been argued that DNA damage is one of the most important drivers of aging.^3,22,23^ A previous report showed that chromodomain helicase DNA-binding protein 4 (CHD4), a key component of the nucleosome remodeling and histone deacetylation (NuRD) complex plays a central role in DNA damage-repair mediated gene silencing in cancer cells.^24^ Therefore, we hypothesized that age-related DNA methylation could also be mediated by DNA damage and its repair process. We used hydrogen peroxide (H_2_O_2_)-treated human fibroblast cells as an aging model. After H_2_O_2_ treatment (100 μM/ 24 h), human fibroblast cells showed a significant increase of senescent markers (Fig. 1d, e). This phenotype is consistent with the senescent gene expression patterns in high-passage number (30–40 passages) cells (Fig. 1d, e and Supplementary Fig. 1f, g). In addition, the increased DNA methylation of Elovl2 was observed in H_2_O_2_-treated cells (Fig. 1f), indicating that DNA methylation occurred in both H_2_O_2_-treated cells and high-passage number cells (Fig. 1d). Next, we examined whether CHD4 could interact with DNA methyltransferases (DNMTs) and chromatin suppression modifiers to mediate abnormal DNA methylation in the H_2_O_2_ treated fibroblast cells. Co-immunoprecipitation assays showed that CHD4, a key component of NuRD complex interacted significantly with DNMTs after H_2_O_2_ treatment (Fig. 1g). Western blotting further showed that the binding of DNMTs to chromatin was promoted by H_2_O_2_ treatment (Fig. 1h, Tight chromatin, Control), but was diminished by CHD4 knockdown (Fig. 1h, Tight chromatin, iCHD4, where the type B nuclear lamin (Lamin B) works as positive control and the cytoplasmic glyceraldehyde 3-phosphate dehydrogenase (GAPDH) works as negative controls), indicating that the H_2_O_2_-induced accumulation of DNMTs to chromatin was mediated, at least partially, by CHD4. Furthermore, we were able to detect endogenous CHD4, γH2a.X and increased 5mC signals at the damage sites 60 min post laser-induced DNA damages, which were dramatically reduced upon CHD4 knockdown (Fig. 1i), indicating that CHD4 plays a central role in DNA damage-mediated DNA methylation during the repair process. Next, we compared the expression of Elovl2 in human fibroblast cells before and after H_2_O_2_ treatment by qPCR. The expression of Elovl2 was significantly decreased after H_2_O_2_ treatment in groups with individual DNMT knockdown, but not with CHD4 knockdown (Supplementary Fig. 1h), indicating that individual knockdown of a single DNMT would not block H_2_O_2_ induced Elovl2 silencing, because other epigenetic modifiers like G9a, which establishes H3K9me3, also can be recruited by CDH4.^25^ On the other hand, knockdown of CHD4 dramatically rescued the expression level of Elovl2 after H_2_O_2_ treatment, which indicated a central role in mediating DNA methylation and further downregulating transcriptional activity of Elovl2. Overall, these results showed that age-related DNA methylation may be mediated by NuRD complex upon DNA damage.

### AI-based 3D protein structure prediction for ELOVL2 protein and its interaction with a substrate

**Fig. 2:**
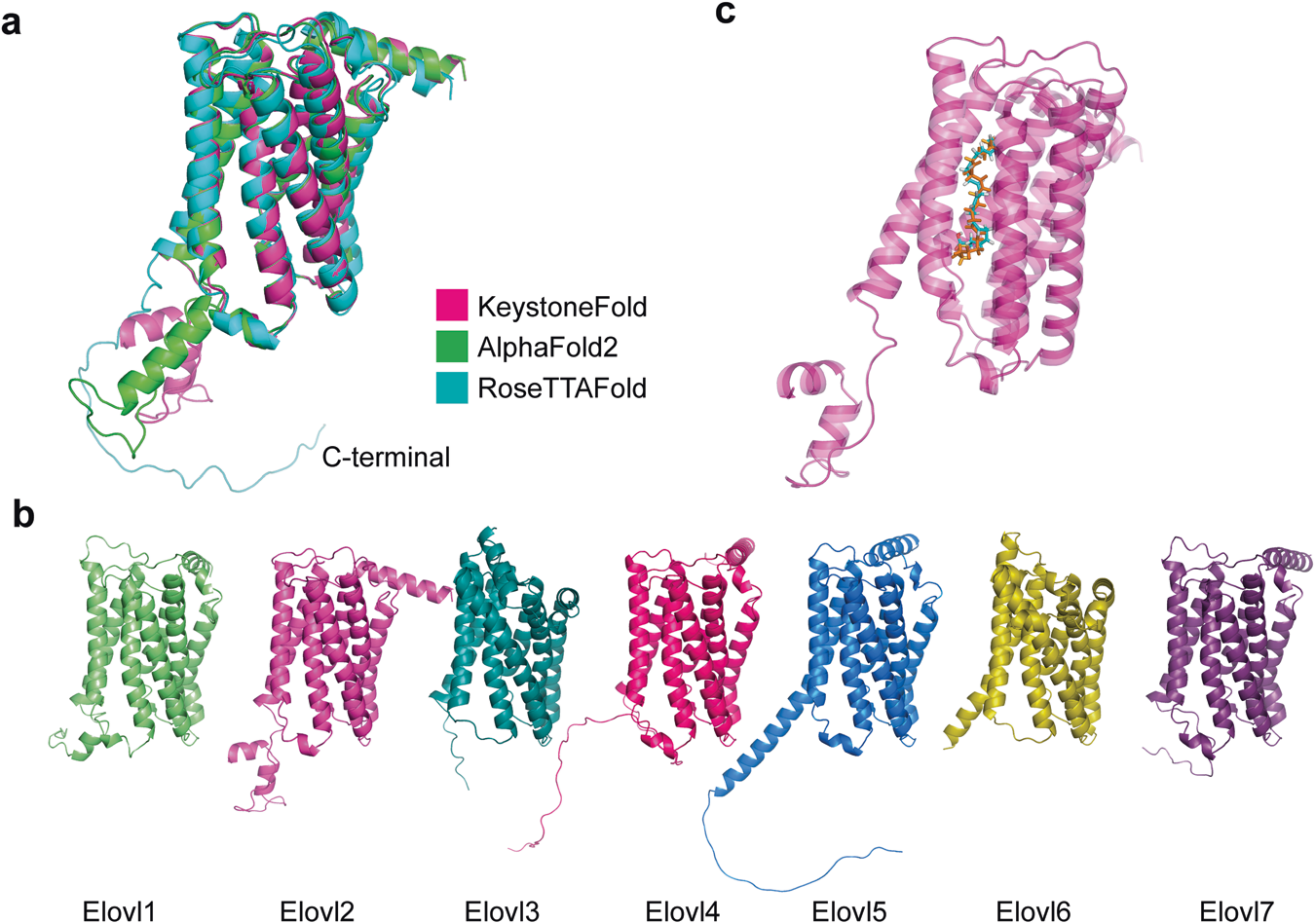
Predicted structures of ELOVLs. **a** Alignment of the structures of ELOVL2 predicted by KeystoneFold, RoseTTAFold, and AlphaFold2. All structures are represented as cartoons and colored according the algorithms used. The main structural difference is in the C-terminal. **b** The structures of ELOVL1-7 predicted by KeystoneFold. All structures are represented as cartoons. **c** The binding mode of lipid substrates PUFAs are predicted by molecular docking (represented as blue and orange sticks)

Currently, there is no complex structure available for ELOVL2 in the PDB database. There are seven members of the ELOVL family, in which the structure of ELOVL7 has been solved.^25^ In order to identify potential common structural features shared among ELOVL proteins and gain broad insights into their functions, we used an AI-based method to predict the 3D protein structures of these proteins, and their complexes with PUFA substrates. The predicted structure of each ELOVL protein by our AI model, KeystoneFold, was very similar to those predicted by RoseTTAFold and AlphaFold2 (Fig. 2a). Our predictions also showed that the seven ELOVL proteins adopted very similar structures (Fig. 2b) as 7 transmembrane domains which form a core catalytic pocket. In order to identify the part of the domain responsible for its substrate binding, we applied molecular dynamics modeling to assess and compare potential docking interactions of PUFAs with ELOVL2 (Fig. 2c). Our AI model predicted a favorable interaction between the central active site tunnel of ELOVL2 and PUFAs.

### Deletion of Elovl2 causes dramatic accelerated aging phenotype in mice

**Fig. 3:**
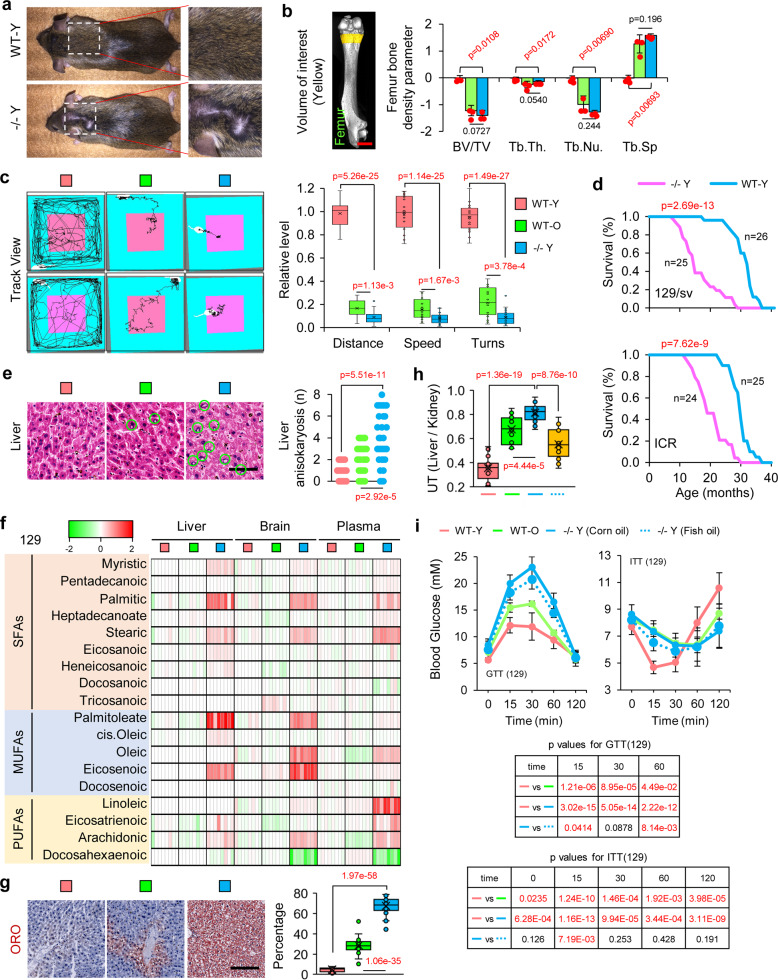
Deletion of Elovl2 caused severely accelerated aging phenotypes and metabolic dysfunction in mice. **a** Hair loss in young (8 month) Elovl2 knockout (−/− Y) but not wild-type (WT-Y) 129/sv mice. **b** Micro-computed tomography (micro-CT) showing bone volume/total volume (BV/TV) and trabecular thickness (Tb. Th.) in the femur of mice (*n* = 3 per group). The WT-O mice are 20-month-old kept in our animal facility with regular diet and living condition. Error bars, SEM. Levels of significance were calculated with two-tailed Student’s *t* test. **c** Open-field test results in different groups of 129/sv mice (*n* = 20 per group). Error bars, SEM. Levels of significance were calculated with two-tailed Student’s *t* test. **d** Survival curve of Wild-type 129/sv and ICR vs Elovl2 knockout mice (WT *n* = 26, −/− *n* = 25 in 129/sv; WT *n* = 25, −/− *n* = 24 in ICR group). **e** Hematoxylin and eosin staining and pathological section analysis of liver tissue. Green cycles show the abnormal structures. Scale bar, 100 µm. For each group 40 slices from five mice were sed for statistical analysis. Levels of significance were calculated with two-tailed Student’s *t* test. **f** Heat-map of fatty acid species in the liver, brain and plasma of 129/sv mice (*n* = 8 per group, fold change in log2 scaled is used). **g** Oil Red O (ORO) staining of liver. Error bars, SEM. Scale bar, 100 µm. For each group 40 slices from five mice were sed for statistical analysis. Levels of significance were calculated with two-tailed Student’s *t* test. **h** Ultrasound test results (*n* = 20 per group). Error bars, SEM. Levels of significance were calculated with two-tailed Student’s *t* test. **i** Glucose tolerance test (GTT) and insulin tolerance test (ITT) results (*n* = 12 per group). Error bars, SEM. Levels of significance were calculated with two-tailed Student’s *t* test

Previous studies on loss of Elovl2 function were mainly restricted to mouse reproductive development and lipid metabolism,^19,26^ however, age-related phenotypes have not been fully studied. To examine the function of Elovl2 in the aging process, we generated Elovl2 knockout mice with CRISPR-Cas9 technology (Supplementary Fig. 2a). In total, we generated 40 Elovl2^+/−^ and 84 Elovl2^−/−^ founder mice. In Elovl2^−/−^ mice, 32 mice were bearing a 59 bp deletion in the 3rd exon that created a stop codon. Western blotting showed that Elovl2 was completely depleted in Elovl2^−/−^ mice (Supplementary Fig. 2b). Our experiments were performed on these 59-bp deletion mice because both Elovl2^+/−^ and Elovl2^−/−^ mice were infertile, irrespective of gender or strain; this was not consistent with the previous report.^19^ This may be caused by different knockout conditions and diet supplement methods. In our study, Stringent Non-PUFA milk and ingredient defined non-PUFA diet was used for the pups or adult mice to prevent the consumption of PUFAs from the food intake. We then examined key aging parameters after 8 months of growth. Elovl2^−/−^ young mice (−/− Y) showed a series of aging-accelerated phenotypes, including hair loss (Fig. 3a), reductions in bone density (Fig. 3b and Supplementary Fig. 2c), endurance (Supplementary Fig. 2d), and muscle strength (Supplementary Fig. 2e). Besides, the open-field behavior test showed that −/− Y mice exhibited reduced exploratory behavior and increased anxiety that were similar to the behavior of wild-type old mice (WT-O, 20-month-old) (Fig. 3c). Furthermore, the Morris water maze test suggested a significant decay in learning and memorizing ability in −/− Y mice (Supplementary Fig. 2f). Moreover, the Elovl2−/− mice show much shorter over all lifespan than wild-type mice, showing much earlier death incidence at age of 10 months in both 129/sv and ICR background. This indicated that Elovl2 deficiency could cause severe shortage of lifespan in Elovl2−/− mouse. Aging-related histopathological phenotypes were also detected in −/− Y and WT-O mice in a local tissue physiolocal level (Fig. 3e and Supplementary Fig. 2g, h). These results demonstrated that lack of Elovl2 in mice lead to a remarkable phenotype in acceleration of aging in multiple aspects.

### Lack of Elovl2 disturbs lipid and energetic metabolism

Next, we investigated whether Elovl2 deficiency could accelerate aging through disturbing metabolism. Considering that Elovl2 plays a critical role in lipid metabolism by functioning as an elongase for long-chain fatty acids from 20: C to 28: C^19^ (Supplementary Fig. 3a), we performed lipidomic analysis. There was a dramatic accumulation of fatty acids containing fewer than 20 carbons, and a trend of deficiency of PUFAs in plasma of both WT-O and −/−Y mice. The accumulation of short-chain fatty acids was more obvious in both WT-o and −/−Y of ICR but not in the 129 strain, this indicated a possible genetic effect may exist. A significant decrease of PUFAs, such as DHA, were found in all liver, brain and plasma of −/−Y, however, a relatively low amount of DHA was still remaining in WT-O. (Fig. 3f and Supplementary Fig. 3b). we noted that the overall fatty acid composition of −/− Y mice was more abnormal than the WT-O mice, which indicates the ELOVL2 remaining function in old mice. Although we did not detect adrenic acid, it is reported that ELOVL2 knockout also increases the content of adrenic acid.^27^ Using Oil red O staining, we observed substantial accumulation of fatty acids in hepatocytes in both WT-O and −/− Y mice (Fig. 3g). Furthermore, a steatohepatitis phenotype was detected by ultrasonography in WT-O and −/− Y mice (Fig. 3h). In clinical practices, these changes could cause hepatic steatosis followed by lipotoxicity^28^ and insulin resistance.^29,30^ Given that, we performed a glucose tolerance test and an insulin tolerance test. We observed dramatic glucose tolerance and insulin resistance phenotypes in the −/− Y mice (Fig. 3i and Supplementary Fig. 3c). This result was consistent with a previous study which reported that the transcriptional activity of Elovl2 had an intimate connection with insulin secretion.^16^ Collectively, these results were indicative of severe metabolic dysfunction in Elovl2-deficient mice.

### A diet supplemented with PUFAs can partially rescue the aging phenotype in Elovl2 knockout mice

Knowing that lack of Elovl2 would diminish the synthesis of PUFAs, we next investigated whether the dietary supplementation of PUFAs could fully rescue the accelerated aging phenotype. In comparison to the control group, dietary supplementation with PUFAs (fish oil) slightly ameliorated fatty acid accumulation in the liver of −/− Y mice and improved physiological glucose metabolic balance, but was insufficient for complete recovery (Fig. 3g, h and Supplementary Fig. 3c). Open-field behavioral test showed that dietary supplementation with PUFAs led to slight improvement of aging phenotype (Supplementary Fig. 3d). Furthermore, the dietary supplementation of PUFAs did not relieve aging-related histopathological phenotypes (Supplementary Fig. 3e). These results indicated that the contribution of lack of Elovl2 to aging did not occur solely through a deficit of nutrient PUFAs.

### Lack of Elovl2 lead to chronic inflammation and adult stem cell exhaustion in mice

Adult stem cells are essential for the maintenance of tissue function and homeostasis. It has been revealed that metabolism play a key role in maintaining an adult stem cell pool.^31^ Recently, Oishi reported that lipid metabolism, particularly PUFAs synthesis, plays a critical role in the immune response.^32^ PUFAs such as DHA and eicosapentaenoic acid, as precursors of inflammation inhibitors, can regulate inflammation.^33,34^ In consideration of that, we hypothesized that −/− Y mice might generate extensively chronic inflammation.

**Fig. 4:**
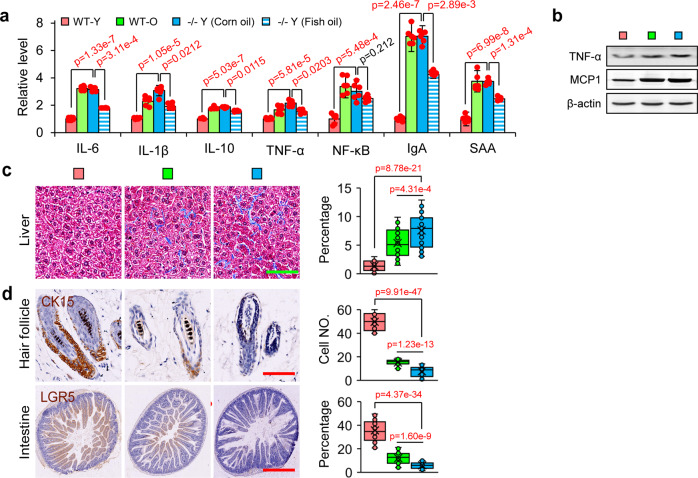
Depletion of Elovl2 led to chronic inflammation, cellular senescence and adult stem cell exhaustion. **a** Inflammatory factors levels in blood measured by ELISA (*n* = 6 per group). Error bars, SEM. Levels of significance were calculated with two-tailed Student’s *t* test. **b** Western blotting of TNF-α and MCP-1 (*n* = 3). **c** Masson’s trichrome staining on liver. Error bars, SEM. Scale bar, 100 µm. For each group 40 slices from five mice were sed for statistical analysis. Levels of significance were calculated with two-tailed Student’s *t* test. **d** Hair follicles and intestines stained with the epithelial progenitor cell markers. Scale bar, 100 µm. Error bars, SEM. For each group 40 slices from five mice were sed for statistical analysis. Levels of significance were calculated with two-tailed Student’s *t* test

Indeed, analysis of blood samples showed a significant increase in the levels of inflammatory factors in both −/− Y and WT-O mice (Fig. 4a and Supplementary Fig. 4a). We also examined the status of inflammation in the liver and found that levels of MCP1 and TNF-α were dramatically increased in WT-O and −/− Y mice (Fig. 4b). It is well-known that chronic inflammation leads to tissue fibrosis and the exhaustion of endogenous stem/progenitor cell reservoirs.^33,34^ In line with these expectations, we observed an increase of fibrosis in the liver (Fig. 4c). Furthermore, a significant loss of the stem cell population in hair follicles (CK15) and in the intestine (LGR5) was detected (Fig. 4d).

Next, we examined the cerebral cortex and hippocampus structure by magnetic resonance imaging, which revealed a dramatic abnormity in −/− Y and WT-O mice (Supplementary Fig. 4b). RNA-Seq analysis also showed a remarkably abnormal expression profile and impaired functional gene expression pattern in the brains of −/− Y mice (Supplementary Fig. 4d, e). These results indicated that failure to resolve endogenous or extrinsic inflammatory stimuli in tissues lack of Elovl2 led to chronic inflammation, stem cells exhaustion, and a loss of tissue function, which in turn resulted in aging phenotypes.

### RNA-seq analysis indicates various metabolic and aging-related pathways were impaired by Elovl2 depletion

**Fig. 5:**
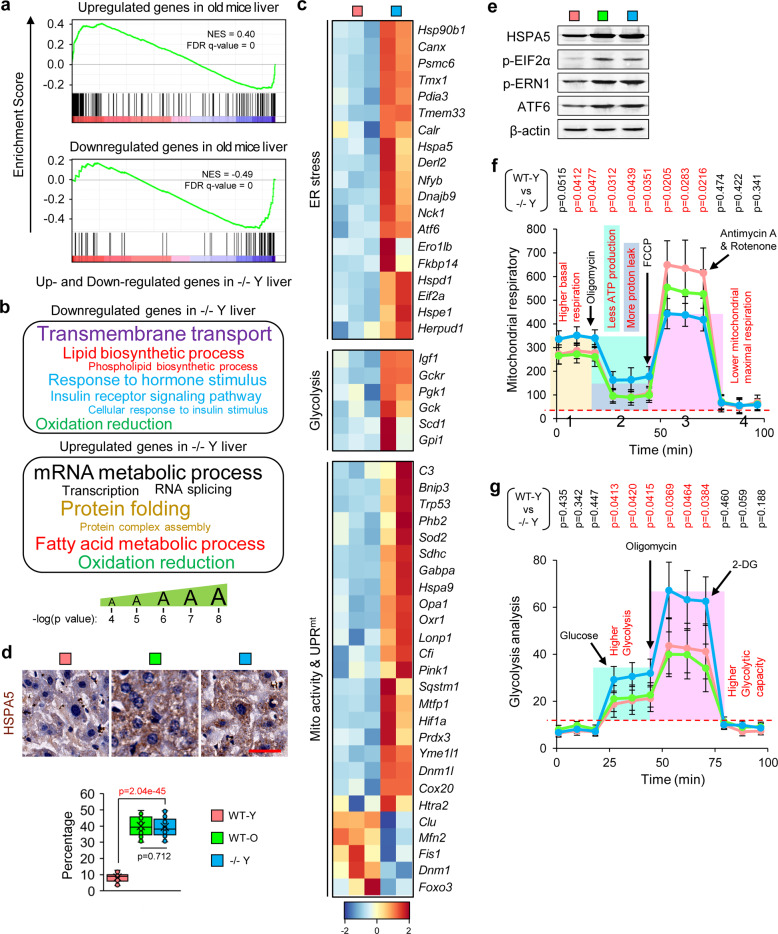
Elovl2 deficiency led to endoplasmic reticulum stress and mitochondrial dysfunction. **a** Enriched gene sets of differentially expressed genes in −/− Y samples. The horizontal axis represents the differentially expressed genes in −/− Y compared to WT-O samples which were ranked as either up- or downregulated in −/− Y and marked in red and blue, respectively. The normalized enrichment score (NES) and false discovery rate (FDR) are marked. **b** The Gene Ontology terms enriched in up -or downregulated genes in −/− Y compared to WT-Y mice. The actual *p* values were shown in Supplementary Table 2. **c** Expression pattern of genes in aging-related pathways of different expression genes in −/− Y mice compared to WT-Y mice (*n* = 3 for WT-Y and *n* = 2 for −/− Y). **d** HSPA5 staining in liver. Error bars, SEM. For each group 40 slices from five mice were sed for statistical analysis. Levels of significance were calculated with two-tailed Student’s *t* test. **e** Western blotting for the markers of ER stress (*n* = 3). **f** The Seahorse XF mitochondrion stress test (*n* = 4). Error bars, SEM. Levels of significance were calculated with one-tailed Student’s *t* test. **g** The Seahorse XF glycolysis test (*n* = 4). Error bars, SEM. Levels of significance were calculated with one-tailed Student’s *t* test

To identify the molecular mechanism responsible for loss of Elovl2 in aging, we performed RNA-Seq on liver and brain samples from WT-Y and −/− Y mice. In comparison to the upregulation of genes in old mice reported in previous studies,^35^ these genes were also enriched in our −/− Y mice (Fig. 5a); the downregulated genes showed a consistent pattern (Fig. 5a). Interestingly, we found that −/− Y mice had a similar expression profile to those on a high fat diet,^36^ which verified that Elovl2 ablation resulted in fatty acid accumulation (Supplementary Fig. 5a). Next, we identified 1867 differentially expressed genes (*p* value <0.05) in the liver of −/− Y mice compared to that of WT-Y mice, with 1084 genes upregulated and 783 genes downregulated. GO enrichment analysis showed that fatty acid/lipid metabolism, cellular responses to insulin stimulus, mitochondrial function, and uncoupled protein response were mis-regulated (Fig. 5b). Notably, we found that the ER stress-associated genes were upregulated (Fig. 5c and Supplementary Fig. 5b), which was consistent with a previous study reported that lipotoxicity could lead to ER stress.^29,30^ It is well known that both lipotoxicity and ER stress can impair mitochondrial function. Indeed, we found that mitochondrial function associated genes, such as those involved in the fatty acid β-oxidation process and insulin receptor signaling pathway, were downregulated, while mitochondrial uncoupled protein response (UPR^mt^)- and glycolysis-associated genes were upregulated (Fig. 5c). Consistent with RNA-Seq data, qPCR also showed the same expression patterns (Supplementary Fig. 5c). In addition, we have analyzed the differentially expressed overlap genes in both brain and liver, we found 121 overlap genes are downregulated and 304 overlap genes are upregulated. Among these upregulated overlap genes, DNA repair cellular response to DNA damage stimulus was presented on the top from Gene Oncology analysis (Supplementary Fig. 5g). This indicated a common aging/senescent status in both brain and liver tissues in ELOVL2 knockout mice.

### Impaired Lipid metabolism causes a stress response in the ER and mitochondrial dysfunction

Mitochondria participate in the aging process in multiple ways, such as energy production, generation of reactive oxygen species (ROS), and UPR^mt^. Also, mitochondria contribute to the changes caused in the cellular senescence state.^37–40^ Therefore, based on our RNA-seq data analysis and clues from previous reports, we hypothesized that lack of Elovl2 promotes aging inducing ER stress and mitochondrial dysfunction.

To verify our hypothesis, we measured ER stress level and mitochondrial function in liver tissues. ER stress markers, such as HSPA5, phosphorylated EIF2α (p-EIF2α), p-ERN1, and ATF6 were upregulated in WT-O and −/− Y mice (Fig. 5d, e). We then used the Agilent Seahorse XF Cell Mito Stress Test to investigate key parameters of mitochondrial function by directly measuring the oxygen consumption rate (OCR) of primary hepatocytes from WT-O and −/− Y mice. For basal respiration, we found that the OCR was significantly increased in −/− Y mice (Fig. 5f, 1), which was not diminished by oligomycin treatment, suggesting an increased incidence of uncoupled respiration (Fig. 5f, 2). Indicating there is hypoxia-mediated mitochondrial dysfunction in −/− Y mice which may also result from superfluous fatty acids.^41,42^

Predictably, after the addition of FCCP, there was a lower level of mitochondrial maximal respiration in −/− Y mice (Fig. 5f, 3). In addition, we found that −/− Y mice showed increased glycolytic activity (Fig. 5g). A switch of metabolism from oxidative phosphorylation to glycolysis, known as the Warburg effect, was observed in both cancerous and senescent cells. It was also identified in our RNA-seq data (Fig. 5c).

One of the consequences of chronic ER stress and mitochondrial dysfunction is oxidative damage at the cellular level. Using varied means of detection, we found that oxidative damage developed in both mitochondria (Supplementary Fig. 5d, MitoSOX for mitochondrial superoxide) and nuclei (Supplementary Fig. 5d, γ-H2AX for DNA oxidative damage), affecting proteins (Supplementary Fig. 5e, AOPP), lipids (Supplementary Fig. 5e, MDA), and RNA (Supplementary Fig. 5e, 8-OHG). As expected, antioxidative enzymes were overactivated (Supplementary Fig. 5e, GSH-PX, CAT, T-SOD, and TAC). Notably, upon such severe oxidative damage, higher cellular senescent markers were detected in −/− Y and WT-O mice (Supplementary Fig. 5f).

In summary, Elovl2 absence could lead to short-chain fatty acid accumulation, ER stress, and mitochondrial dysfunction at cellular level.

### Elovl2 deficiency in human RPE cells induces an AMD phenotype

**Fig. 6:**
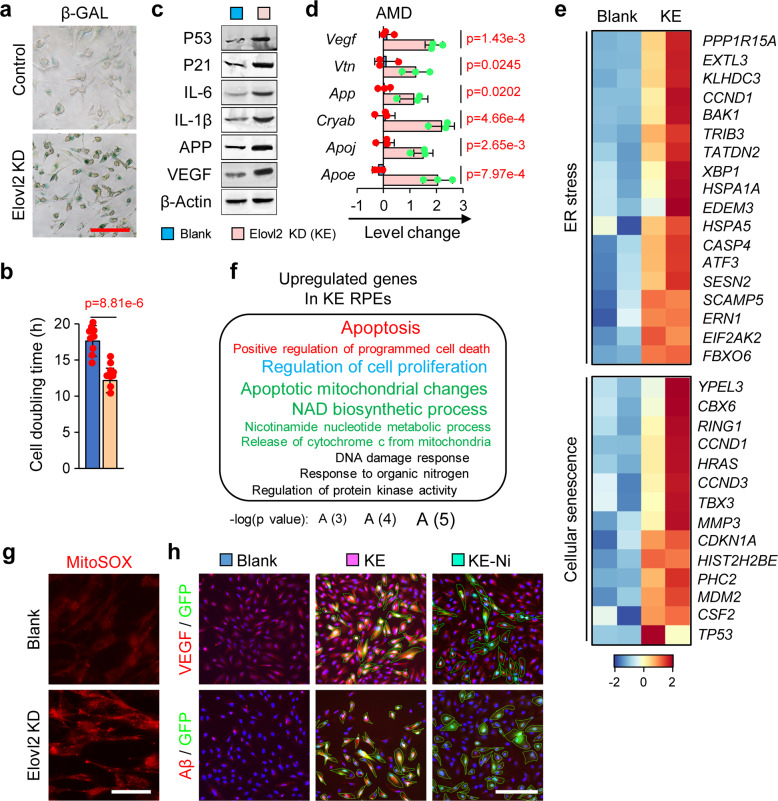
AMD phenotype induced by the depletion of Elovl2 in human RPE cells. **a** β-galactosidase staining on human RPE cells with no treatment (Control) or Elovl2 knockdown (Elovl2 KD) (*n* = 3). Scale bar, 100 µm. **b** Cell doubling time analysis (*n* = 9). Error bars, SEM. *p < 0.05. Levels of significance were calculated with two-tailed Student’s *t* test. Western blotting (**c**) and qPCR (**d**) of senescence and AMD marker in blank and KE cells (*n* = 3). *p < 0.05. Levels of significance were calculated with two-tailed Student’s *t* test. **e** The expression pattern of genes in ER stress- and cellular senescence-related pathways (*n* = 2). **f** The Gene Ontology terms enriched in upregulated genes in KE RPE cells. **g** mitoSOX staining results (*n* = 3). Scale bar, 100 µm. **h** Immunofluorescence of blank and KE RPE cells treated with nicotinamide (Ni) with VEGF and Aβ antibodies (*n* = 3). Scale bar, 100 µm

Our previous results showed that the depletion of Elovl2 lead to a degenerative phenotype of the central nervous system in mice. We hypothesize a potential degenerative effect might also be induced in optic nerve system by ELOVL2 deficiency. Age-related macular degeneration (AMD) is an eye disease that can blur central vision. It happens when aging causes damage to the macula. In our previous study, we have found AMD like phenotypes in the eyes of Elovl2 knockout mice showing increase of cell senescent signal in different layers of retina and loss of visual function (data not shown). These phenotypes are consistent with a recently published paper by Chen et al. in which AMD phenotype was confirmed by drusen and other markers in Elovl2 mutant mice.^43^ Given that, we next investigated whether Elovl2 ablation could result in an AMD phenotype in human cells. We developed Elovl2 knockdown RPE cell lines (KE) via the lentivirus delivery of shRNAs to human primary RPE cells (generated from healthy donors) (Supplementary Fig. 6a, b). As expected, the knockdown of Elovl2 resulted in cellular senescence (Fig. 6a) and impaired proliferation (Fig. 6b).^37^ We also detected the upregulation of SASP markers, including P53, P21, IL-1b and IL-6^39^ in KE cells (Fig. 6c). Numerous senescence and AMD markers were found to be increased at both the RNA and protein level in KE cells (Fig. 6c). These results indicated that an AMD model, with an increased SASP phenotype, can be generated by the deficiency of Elovl2 in human RPE cells.

As mentioned above, lack of Elovl2 led to fatty acid accumulation, which triggered chronic ER stress and mitochondrial dysfunction. Thus, we hypothesized that these impairments also occur in the Elovl2 deficiency-induced human AMD model. Indeed, from RNA-seq analysis, we found a dramatic increase in chronic ER stress and cellular senescence in KE cells (Fig. 6e and Supplementary Fig. 6c). Moreover, GO term analysis showed mitochondrial dysfunction in KE cells; genes associated with mitochondrial function, such as those controlling the NAD biosynthetic process and apoptotic mitochondrial changes, were dysregulated (Fig. 6f and Supplementary Fig. 6d). Furthermore, we detected the accumulation of oxidative damage in the mitochondria (Fig. 6g), which reflected severe oxidative damage in KE cells. In both the mouse and human cell models, Elovl2 deficiency led to an increase in oxidative damage caused by chronic ER stress and mitochondrial dysfunction.

Mitochondrial abnormalities are an early driver of neuronal dysfunction. it’s been reported that restoration of mitochondrial function by NAD^+^ precursor nicotinamide (vitamin B3) was protective both prophylactically and as an intervention against Glaucoma, a neurodegenerative diseases that cause vision loss, especially in the elderly.^40^ To further confirm whether restoration of mitochondria can rescue AMD phenotype in Elovl2 Knockdown RPE cells, we treat the Elovl2 knockdown RPE cells with nicotinamide (Ni). Interestingly, treatment with Ni during the culturing of KE cells dramatically reversed the abnormal expression of cellular senescence genes and reduced mitochondrial dysfunction, thus causing a remarkable reduction of AMD markers (Fig. 6h). Together, these results indicated that restoration of mitochondria function can ameliorate the AMD phenotype caused by Elovl2 depletion.

## Discussion

Epigenetic alteration is one of the hallmarks of aging. The integral relationship between aging and DNA methylation levels was established in the late 1960s; however, it was only recently that correlation between DNA methylation in several hundred CpG sites and biological age were precisely estabolished.^6,11^ We and others have shown that the DNA methylation of specific gene loci can be used as an accurate biomarker of biological aging in human and mice.^4,11,15,16^ The functional consequences of DNA methylation on these CpG sites have become in many discussions and speculations. In our previous report, we identified a series of aging markers; some of these marker genes are involved in lipid metabolism. Intriguingly, Elovl2, a gene which functions as a master control of lipid metabolism and is strongly associated with diabetes, shows the most relevance to aging.^4,11,15,16^ Slieker et al. claimed that ELOVL2 is a unique tissue-independent age-associated DNA methylation marker,^15^ however, a subsequent report showed that ELOVL2 is not a unique universal aging marker and that there are many more CpG sites/genes that are consistently altered with age across many different cell/tissue types. Some of them map to genes in Wnt and glutamate receptor signaling pathways and are altered with age across at least ten different cell/tissue types.^17^ This intriguing observation also indicate a potential important role of Wnt pathway in aging since Wnt pathway intimately contribute to maintaining adult stem cell pool during aging. Further study needs to be done in this emerging field.

The mechanism of how aging-related DNA methylation is mediated remains unclear. Cellular damages play a key role in initiating epigenetic gene silencing.^3,22,23^ The transcription repressors can be immediately recruited to silence transcription on the site soon after DNA damage occurs.^24^ Chromatin silencing is required to ensure the damage repair process. Meanwhile, the subsequent abnormal DNA methylation retained after this process. Here we showed that NuRD complex plays a key role in the mediation of DNA methylation on these age-related sites during cellular oxidative damage or following DNA double-/single-strand breaks. However, it still needs future study to learn the mechanism of random DNA damage induced DNA methylation.

Elovl2, an important aging marker, plays a critical role in very long-chain fatty acid elongation. Previous studies show that Elovl2 is also associated with diabetes by genome wide association studies in mouse models.^16^ Supporting the notion that Elovl2 is a crucial link between metabolism and aging. Here, we investigated whether Elovl2 played a functional role in aging first in a genetically modified mouse model. Although the lifespan of human and mouse is largely different (particularly the maximum lifespan differs dramatically), the shape of the theoretical lifespan curves, often considered representative of the health of the organism, is remarkably consistent across species.^44^ Several review papers have nicely showed the conserved phenotype and mechanism of ageing shared in both human and mouse, some of these are even remarkably consistent across species including yeast, worm and flies. Indeed, Lopez-Otin et al. have categorized these conserved features of aging into a set of 9 “hallmarks of aging”,^45^ many of which span the evolutionary distance from yeast to human. The 9 identified hallmarks of aging are as follows: genomic instability, mitochondrial dysfunction, deregulated nutrient sensing, loss of proteostasis, epigenetic alterations, cellular senescence, stem cell exhaustion, altered intracellular communication, and telomere attrition. It is much valuable to take this advantage to study the aging mechanism in animal models and reach out to human. In this study we used CRISPR-Cas9 to generate Elovl2 knockout mice. In contrast to previous report, in our study we found dramatic aging acceleration phenotype and many of features of nine hallmarks of aging in the Elovl2 knockout mice, suggesting its crucial role in the aging process. In addition, the structure of Elovl2 and other family members have not been fully revealed. With the rapid advancement of AI application in the structural biology, we used our KeystoneFold model, together with RoseTTAFold, and AlphaFold2 method, to predict the structure of ELOVL2. The successful description of protein structure of ELOVL2 and other ELOVL family could help us better understand the lipid synthesis process in different perspective.

Lipid synthesis is based on the normal function of ER, the location of Elovl2. Apart from lipid synthesis, the ER also performs important functions related to the synthesis, folding, and transport of proteins. It has previously been reported that the accumulation of free fatty acids in the ER would damage ER function, resulting in an increased incidence of unfolded or misfolded protein load and chronic ER stress. A reduction in PUFA synthesis upon Elovl2 ablation leads to a compensatory increase in fatty acid synthesis which can affect cellular metabolic homeostasis through the accumulation of fatty acid precursors for PUFAs in the ER and changes in mitochondrial energy metabolism. ER stress is also related to insulin resistance and mitochondrial dysfunction (Supplementary Fig. 7).

Mitochondria are the powerhouse of the cell and play a prominent role in producing energy through respiration and regulating cellular metabolism.^38,39^ Elovl2 deficiency induced a switch in metabolism from the tri-carboxylic acid cycle to glycolysis, an effect which produces more reactive oxidative species (ROS), causes oxidative stress in cells, tissues, and organs, and also act as a messenger for inflammatory responses. In addition, PUFAs are essential in the resolution of inflammation. In addition to that, there was a dramatic accumulation of fatty acids upon Elovl2 knockout, including arachidonic acid. As accumulation of arachidonic acid might also contribute to inflammation for its being used for PGE2 generation, PGE2 may be involved in inflammation upon Elovl2 knockout. We would study it in the future. In the context of Elovl2 knockout, there were notable increases in physiological inflammation, cellular senescence and adult stem cell exhaustion in a range of tissues (Supplementary Fig. 7). In line with the idea that these physiological changes contribute to aging phenotype, we found an AMD like phenotype in the eye of Elovl2 knockout mice indicated by increase of cell senescent signal in different layers of retina, destruction of RPE layer^46^ and loss of visual function. These phenotypes are consistent with a recently published paper by Chen et al. in which AMD phenotype was confirmed by drusen and other markers in Elovl2 mutant mice.^43^ Next, we confirmed that this epigenetic-metabolism-aging axis also exists in human. Increased levels of AMD markers were detected with similar accumulations of ER stress, mitochondrial dysfunction, and cellular senescence in human primary RPE cells. The restoration of mitochondrial activity by nicotinamide dramatically reduced the levels of AMD markers in Elovl2 knockdown human RPE cells.

Our result shows that epigenetic changes alter metabolism during aging and provide a better understanding of the molecular mechanisms underlying the epigenetic and metabolic aging process. Metabolic dysfunction is an effector of age-related epigenetic alterations. Meanwhile, metabolic dysfunction can also be a mediator that causes epigenetic changes reciprocally. The mechanism of how metabolism regulated age-related epigenetic alteration still needs to be further studied.

## Materials and methods

More information about experimental procedures is provided in the Supplementary Materials online, Materials and Methods.

### Animal care and use

All mouse experiments were performed in accordance with the Guidelines for the Use of Animals in Research issued by the Institute of Zoology, Chinese Academy of Sciences. Mice were purchased from Beijing Vital River Laboratory and housed in the animal facilities of the Institute of Zoology.

### Aging marker genes analysis

The data were produced in our previous work.^11^ Briefly, Genomic DNA was extracted from the whole blood of participants. Methylation fraction values for the autosomal chromosomes were measured with the Illumina Infinium HumanMethylation450 BeadChip. The data were analyzed by BeadStudio software v3.2. Annotation enrichment tests for CpG islands’ Methylation marker were performed with the two-sided Fisher’s exact test. The aging model were described in our previous work.^11^ The regression coefficient was calculated based this model. The covariates gender, BMI, diabetes status, ethnicity, and batch were included in the model and were exempted from penalization (regularization). *p* values are based on a least-squares model built with the same terms and drop-one F tests.

### Protein structure prediction

We developed an attention mechanism-based deep learning model called KeystoneFold inspired by AlphaFolder 2^20^ and RoseTTAFold architecture.^21^ Briefly, multiple sequence alignments (MSAs) were build using HHblits in the HH-suite3 package^47^ to generate features for the proteins. The input sequence feature matrixes were converted to three-dimensional matrixes in the hidden layers. The attention architectures inspired by Transformer were used to capture long-range sequential context of residues from given MSA features. Full-atom structure models were generated based on gradient-based folding using pyRosetta.^48^ The residue-wise Cɑ-lDDT scoring function^49^ was used to select final models from all the sampled structures. Substrate docking of Elovl2 was prepared and performed using Discovery Studio 3.1.

### Experimental model and subject details

#### Elovl2***+***/− and −/− Mouse

All mouse experiments were performed in accordance with ARRIVE guidelines and regulations. Zygotes were collected from 6-week-old ICR superovulated female mice crossed with ICR males, at post human chorionic gonadotropin (phCG) injection 21 h. Cas9 mRNA and sgRNAs targeting Elovl2 exon 3 (Supplementary Fig. 1, 20 ng/μl for each) were injected at phCG 25 h. The Cas9 mRNA was in vitro transcribed with mMESSAGEmMACHINE^®^ T7 ULTRA Kit (Ambion, AMB1345-5), and sgRNAs were synthesized by in vitro transcription using MEGAshortscript™ Kit (Ambion, AM1354).

#### Cell culture and drug treatment

Human primary RPE cells were derived from post mortem tissues from an eyebank and were exempted from ethics approval. The human RPE cells were primarily derived from healthy donors. The human fibroblast cells were purchased from ATCC (WI-38, ATCC, CCL-75) Human fibroblasts were maintained in Dulbecco’s Modified Eagle Medium (DMEM) supplemented with 10% fetal bovine serum and 50 U/ml penicillin-streptomycin. Human RPE cells were cultured with DMEM/F-12 (1:1) supplemented with 10% fetal bovine serum and 50 U/ml penicillin- streptomycin.

For Elovl2 knockdown, a lentiviral vector (pLL3.7-shElovl2) exogenously expressed shRNA (5′- TGGTGGTACTATTTCTCCAAA-3′) was made and transfected into 293T cells (ATCC) using Lipofectamin 3000 reagent (Thermo Fisher, L3000008) to obtain lentivirus. To build the Elovl2- knockdown cell line, human RPE cells (seeded a night ahead in a concentration of 5 × 10^5^ cells/well) in a 6-well plate were infected with the lentivirus containing Elovl2-shRNA for 24 h, followed by 24 h of equilibration. Drug treatments were performed at the time of 48 h post-infection. To rescue the Elovl2 knockdown cells, 10 μg/ml curcumin (Sigma, C7727) or 5 mM nicotinamide (Sigma, N0636) or DMSO were added in the cell culture medium and the cells were treated for 48 h. H_2_O_2_ treatments were performed on similarly confluent fibroblast cells to avoid variability of H_2_O_2_ effect since H_2_O_2_ toxicity is inversely related to cell density. Briefly, cells on the 2nd day of passage were treated with H_2_O_2_ at the concentration of 100 µM in a single dose for 24 h and then split at a ratio of 1:3 in fresh DMEM with 10% FBS medium followed by SA–β-gal staining.

## Supplementary information

Supplemental Material

## Data Availability

The sequencing data have been deposited in Genome Sequence Archive of Beijing Institute of Genomics, Chinese Academy of Sciences (http://gsa.big.ac.cn/). The accession number for mouse and human data reported in this project are CRA002140 and CRA002141. The customized codes for predicting 3D protein structure and molecular interaction are available upon reasonable request to the corresponding authors.
