# Supplementary material for: Lipid metabolism dysfunction induced by age-dependent DNA methylation accelerates aging
Source: Signal Transduct Target Ther. 2022 May 25;7:162. doi: 10.1038/s41392-022-00964-6 (PMC9130224; doi:10.1038/s41392-022-00964-6)
Supplement: Supplementary file 1 — Supplemental Material [file 41392_2022_964_MOESM1_ESM.docx]

Supplementary Materials for

lipid metabolism dysfunction induced by age-dependent DNA methylation accelerates aging

Xin Li^1,2,3†^, Jiaqiang Wang^1†^, LeYun Wang^1†^, Yuanxu Gao^4,5†^, Guihai Feng^1^, Gen Li^6^,Jun Zou^5^, Meixin Yu^6^, Yu Fei Li^1^, Chao Liu^1^, Xue wei Yuan^1^, Ling Zhao^7^, Hong Ouyang^7^, Jian-Kang Zhu^8*^, Wei Li^1*^, Qi Zhou^1*^ and Kang Zhang^3*^

Correspondence to: kang.zhang@gmail.com; liwei@ioz.ac.cn; qzhou@ioz.ac.cn; jkzhu@sibs.ac.cn;

**This PDF file includes:**

Materials and Methods

References

Figures. S1 to S8

Tables S1 to S2

Materials and Methods

MSA and sequence embedding

To generate features for the proteins, we used HHblits in the HH-suite3 package^1^ to build multiple sequence alignments (MSAs). HHblits is an iterative sequence search tool based on hidden Markov models^2^. It could sensitively and accurately find homologous sequences from protein sequence databases and then build MSAs. We initially generated one MSAs for each protein sequence by running HHblits on the UniRef30 (released in June 2020) and BFD sequence databases with 4 iterations. The E value cutoff iteratively set to 1×10^-30^, 1×10^-10^, 1×10^-6^, 1×10^-3^. the resulting MSA has at least 2000 sequences with 75% coverage or 5000 sequences with 50% coverage (both at 90% sequence identity cutoff). From each individual MSA, we derived sequential features that include sequence profile and secondary structure prediction by PSIPRED^3^. Template structures were obtained by searching the generated MSA against the PDB100 database with HHsearch.

The input MSAs were represented as m×n matrixes, where m corresponds to the number of sequences in the MSAs, and n is the residue position length in the aligned sequence. The individual amino acids and gaps of the input MSAs were tokenized as 21 characters for further processing. The tokenized matrixes were mapped to vectors through an embedding layer. Pairwise features including positional similarity and alignment confidence were calculated by extracting residue pairwise distances.

AI-based structure prediction

We developed an attention mechanism-based deep learning model called KeystoneFold inspired by AlphaFolder 2^4^ and RoseTTAFold architecture^5^. The input sequence feature matrixes were converted to three-dimensional matrixes in the hidden layers. The attention architectures inspired by Transformer were used to capture long-range sequential context of residues from given MSA features. The outer product can capture the correlation information between two residues. The attention mechanism is suitable for protein structure prediction as it could efficiently learn the relationship of residue pairs distant in sequence. The attention layers provide an efficient MSAs representation. The hidden layer outputs were then fed into a transformer-based architecture followed by a linear transformation, that were employed to generate initial Cartesian coordinates of protein backbone atoms. Multiple SE(3)-Transformer layers^6^ were used to refine given 3D coordinates based on original and updated MSA and pairwise features. The average of transposed and untransposed feature maps was used to ensure the symmetry the network predictions. Predicting protein structure with long sequence can exceed the memory of a single GPU. Full-atom structure models were generated based on gradient-based folding using pyRosetta^7^. The residue-wise Cɑ-lDDT scoring function^8^ was used to select final models from all the sampled structures.

Substrate docking

The protonation states of ELOVL2 were assigned using the "Calculate Protein Ionization and Residue pK" module of Discovery Studio 3.1 to correspond to pH 7.0, optimizing the rotameric states for histidine, asparagine, and glutamine residues. The protein model of ELOVL2 was prepared for docking, minimizing, and optimizing hydrogen placement and side chain atoms with CHARMM. Docking was performed using the "Docking Ligands (LigandFit)" module of Discovery Studio 3.1. The docking sites and spheres were generated automatically and then manually inspected and modified where necessary to achieve a more uniform distribution across the binding site. The docking scores LigScore1^9^, LigScore2^9^, PLP1^10^, PLP2^10^, and PMF^11^ were used to relatively ranking of the binding of compounds.

RNA extraction, reverse transcription, and qPCR

RNA was extracted by RNeasy Mini Kit (QIAGEN, 74104) and the RNase-Free DNase Set (QIAGEN, 79254) was used to ensure no DNA contamination. Reverse transcription was performed by High Capacity cDNA Reverse Transcription Kit (ABI, 4368814). qPCR was performed by Power SYBR® Green PCR Master Mix (ABI, 4367659). All primers were designed using PrimerPremier5 (Table S1), and synthesized from Integrated DNA Technologies.

***Methylated DNA immunoprecipitation (MeDip)***

MeDip assays were performed on human fibroblast cells with the Nuclear Complex Co-IP kit (Active Motif, 54001) following instructions of manufacturer. Briefly, cells were lysed in hypotonic buffer and the nuclear extracts (tight chromatin) were gained via centrifugation. Extracts were digested with enzymatic shearing cocktails and incubate overnight with anti-5-methylcytosine (5-mC) antibody (Abcam, ab214727) at 4℃ following by the pull-down with protein A/G magnetic beads (Thermo, 88802). The pull-down products were washed, denatured and eluted according to the manufacturer’s instruction. The MeDip results were then analyzed by qPCR with primers targeting CpG islands of human ELOVL2 (Table S1).

***Bisulfite sequencing analysis for DNA methylation***

Genomic DNA was extracted from brain, liver and testis of mice in different group using the Universal Genomic DNA Extraction Kit (Takara). Then the DNA was treatment with sodium bisulfite to convert all unmethylated cytosine to uracil using EZ DNA Methylation-GoldTM Kit (Zymo Research, Orange, CA, USA) according to the manufacturer’s protocol. Subsequently, nested PCR amplifications of bisulfite-treated DNA were performed using primers shown in Table S1 with Hot Start TaqTM Polymerase (TaKaRa) in 20 μL reaction volume with a thermo profile of 98 °C for 5 min; 94 °C for 30 s, 60 °C for 30 sec, 72 °C for 30 sec, for 40 cycles; followed by 72 °C for 10 min. We used 2 μL of products from the first amplification reaction in the second PCR reaction. The amplified products were verified by electrophoresis on a 1% agarose gel and purified using the Agarose Gel DNA Purification Kit (Takara). Purified fragments were cloned into the pEASY-T1 Vectors (TransGen). The positive clones confirmed by PCR were sequenced by BGI compony. Only sequencing results with more than 95% cytosine conversion were selected for DNA methylation analysis.

***Induction of localized DNA damage by lasers***

Two-photon 450-nm laser irradiation was carried out following a previously described method^12^. Briefly, cells were grown on gridded glass-bottom culture dishes (MatTek) until nearly 50% confluent.

The nuclear were stained with 10 ng/ml Hoechst 33258 for 30 min at 37 °C in 5% CO_2_. The two-photon 450-nm laser beam was focused through a 63× objective to induce local nuclear damage at 100% power. All laser irradiations were performed at 37 °C in a stage warmer.

***IF staining after laser irradiation***

Detailed procedure was described in the following paper: Xia, L. et al. CHD4 Has Oncogenic Functions in Initiating and Maintaining Epigenetic Suppression of Multiple Tumor Suppressor Genes. Cancer Cell 31, 653-668 e657, doi:10.1016/j.ccell.2017.04.005 (2017). Briefly, Fixed cells were permeabilized with a PBS solution containing 0.5% Triton X-100 on ice for 10 min. Then, cells were incubated at 37 °C for 1 hr with anti-γH2AX (Millipore, 05-636; Santa cruz, sc-101696), anti-CHD4 and anti-5mc (Diagenode, C15200081). Cells were incubated with corresponding secondary antibodies. After washing, they were mounted using ProLong Gold antifade reagent with DAPI (Invitrogen). Cells (n = 30) were examined for each experimental point.

***Beta-galactosidase staining***

SA–β-gal staining was done using a SA–β-gal staining kit (catalog no. 9860; Cell Signaling Technology, Danvers, MA, USA) according to the manufacturer's instructions. Briefly,1.Remove growth media from the cells. 2. Rinse the plate one time with 1X PBS (2 ml or a 35 mm well plate, or match volume of media) 3. Add 1 ml of 1X Fixative Solution to each 35 mm well. Allow cells to fix for 10-15 min at room temperature. 4. Rinse the plate two times with 1X PBS 5. Add 1 ml of the β-Galactosidase Staining Solution to each 35 mm well 6. Incubate the plate at 37°C at least overnight in a dry incubator (without CO_2_). 7. While the β-galactosidase is still on the plate, check the cells under a microscope (200X total magnification) for the development of blue color. 8. Taking images and for long- term storage of the plates, remove the β-Galactosidase staining solution and overlay the cells with 70% glycerol. Store at 4°C.

***ELISA and Chemiluminescence substrate detection***

ELISA of IL-6 was performed using IL-6 Mouse ELISA Kit (Invitrogen). ELISA of IL-1β was performed using IL-1 beta Mouse ELISA Kit (Invitrogen). ELISA of IL-10 was performed using Mouse IL-10 ELISA Kit (Abcam). ELISA of TNFα was performed using TNF alpha Mouse ELISA Kit (Invitrogen). ELISA of NF-κB was performed using NF kappaB p65 ELISA Kit (Abcam). ELISA of IgA was performed using IgA Mouse ELISA Kit (Invitrogen). ELISA of SAA was performed using SAA Mouse ELISA Kit (Invitrogen). The oxidative damage affecting proteins was measured via Advanced Oxidation Protein Products (AOPP) Assay (CELL BIOLABS). The oxidative damage affecting lipids was measured via Lipid Peroxidation (MDA) Assay Kit (Abcam). The oxidative damage affecting RNA was measured via OxiSelect™ Oxidative RNA Damage ELISA Kit (8-OHG Quantitation) (CELL BIOLABS). The level of GSH-PX was measured by Glutathione Peroxidase (GSH-Px) Activity Assay Kit (Elabscience). The level of CAT was measured by Catalase (CAT) Activity Assay Kit (Elabscience). The level of T-SOD was measured by Total Superoxide Dismutase (T-SOD) Activity Assay Kit (Hydroxylamine Method) (Elabscience). The level of T-AOC was measured by Total Antioxidant Capacity (T-AOC) Colorimetric Assay Kit (Elabscience).

Western blot

Fresh tissue suspension was obtained by mechanical trituration. Human fibroblast cells and human RPE cells were collected and lysed in Pierce IP Lysis Buffer (Pierce, 87787), supplied with Protease Inhibitor Cocktail (Pierce, 78441) and sodium orthovanadate (Sigma, S6508) on ice for 30 min. After 13,000 g centrifugation for 10 min at 4℃, supernatants were collected and mixed with 30 μL sample buffer (10 mL; 1.25 mL 0.5 M-pH 6.8-Tris-HCl, 2.5 mL glycerin, 2 mL 10% SDS, 200 μL 0.5% bromophenol blue, 3.55 mL H_2_O, and 0.5 mL β-mercaptoethanol) and incubated for 5 minutes in boiling water. The samples were separated on SDS-PAGE with a 5% stacking gel (10 mL; 5.7 mL ddH_2_O, 2.5 mL 1.5M pH 6.8 Tris-HCl, 1.7 mL 30% acrylamide (acryl:bis acryl = 29:1), 100 μL 10% SDS, 50 μL 10% ammonium persulfate, and 10 μL TEMED) and a 10% separating gel (10 mL; 4.1 mL ddH_2_O, 2.5 mL 1.5 M pH 8.8 Tris-HCl, 3.3 mL 30% acrylamide (acryl:bis acryl = 29:1), 100 μL 10% SDS, 50 μL 10% ammonium persulfate, and 5 μL TEMED) at 100 V for 1h, and then electrophoretically transferred onto a nitrocellulose membrane at 200 mA for 1h at 4°C. Membranes were blocked in TBST buffer (10 mM Tris, 150 mM NaCl, 0.1% Tween 20, pH 7.4) containing 3% BSA (Sigma, B2064), for 1h at RT and then incubated with primary antibody, diluted in TBST containing 1% BSA, overnight at 4°C. After three washes for 10 minutes each in TBST, the membrane was incubated for 1 h at RT with the secondary antibody diluted in TBST. After three washes for 10 minutes each, the signals were detected using ECL and films.

***Tight Chromatin and Whole Cell Isolation***

Co-immunoprecipitation

Co-immunoprecipitation of chromatin bound proteins was performed on human fibroblast cells and human RPE cells with the Nuclear Complex Co-IP kit (Active Motif, 54001) following instructions of manufacturer and detailed methods were adapted from published paper^25^. Briefly, cells were collected and pellets were sequentially washed in CEBN buffer [10 mM HEPES pH 7.8, 10 mM KCl, 1.5 mM MgCl2, 0.34 M sucrose, 10% glycerol, 0.2% NP-40, 1× protease inhibitor cocktail (Thermo Scientific), 1× phosphatase Inhibitor cocktail (Sigma), N-ethyl-maleimide (Sigma)], CEB buffer (CEBN buffer without NP-40), soluble nuclear buffer (3 mM EDTA, 0.2 mM EGTA, inhibitors), and 0.45MNaCl buffer (50mMTris pH 8.0, 0.05% NP40, 0.45MNaCl, inhibitors). The remaining pellet was lysed using a Qiashredder (Qiagen) and referred to as tight chromatin. Whole cell extracts were prepared from cell pellets before the tight chromatin isolation. GAPDH and LaminB immunoblotting serve as cytoplasmic and nuclear controls, respectively.cells were lysed in hypotonic buffer and the nuclear extracts (tight chromatin) were digested with enzymatic shearing cocktails and incubate overnight with anti-CHD4 antibody (Sigma, SAB4200107) at 4℃ following by the pull-down with protein A/G magnetic beads (Thermo, 88802). The pull-down products were washed, denatured and eluted according to the manufacturer’s instruction.

Micro-Computed Tomography Bone Density Analysis

Micro-CT was performed using the Inveon MM system (Siemens, Munich, Germany). Images with 8.82 μm pixel size were acquired under 60 kV of voltage, 300 μA of current and 1,500 ms of exposure time during the 360° rotational step. 2000 slices of images with voxel size of 8.82 μm × 8.82 μm × 8.82 μm were acquired. 3D reconstruction was performed using multimodal 3D visualization software (Inveon Research Workplace, SIEMENS, Munich, Germany).

Open field test

The apparatus consisted of a square-shaped arena (600 × 600 mm^2^, length × width) constructed by blue plastic, and illuminated evenly at 15 lux^50^. Test mice were placed facing the center of one wall and allowed to explore the apparatus for 10 min. The open field was subdivided into two virtual concentric squares (center region and all region). The distance and the velocity spent in all regions were calculated.

Morris water maze

The water maze was built with a black tank (2 m diameter) fill with water at room temperature. During the training period, mice were trained to find a fixed platform submerged at constant positions 15 cm below the water surface in one of the quadrant. The mice were placed at four settled spots in the tank and allowed to find a foothold. If a mouse failed to reach the hidden platform in 90 s, it was led to it manually and stayed for 15 s. The training of mice was given for 5 days with 2 consecutive trials per day. The natant trajectory was recorded and analyzed with SMART image system to calculate the path length, swim velocity and number of turns that mice made. For the spatial learning evaluation, difference between the path length of day1 and day 5 was compared. For the spatial memory ability test, the platform was removed from the tank and the mice were released and freely swim in the maze for 90 s. The path length, swim velocity and turn numbers were measured.

Accelerating rotarod test

The test was performed with Ugo Basile system following instruction of manufacturer. Briefly, mice were placed on a rotating rod that rotated from 4 to 40 r.p.m. for 5 min. The time until falling off or losing balance was recorded. For three consecutive days, each mouse was tested for three trials per day with 30 min interval between trials.

Grip strength test

The grip strength test was performed on mice using the TreadScan grip strength measuring system. The mice were allowed to grasp a sensing bar attached to a force strength meter. After reaching the bar with both paws symmetrically, the mice were gently pulled away until the grasp broke. The mean value in five consecutive trails was taken as the score. Results were normalized with body weight (g).

Ultrasonic detection of fatty liver

Ultrasonic scans were acquired with an HDI 5000 scanner (Philips Medical Systems, Bothell, WA), using a broad-bandwidth phased array transducer (2 to 5 MHz). The US images were acquired before biopsy needle insertion; the precise biopsy site was selected based on these images. Images were acquired with the same presetting of the echographic equipment, i.e., imaging probe, gain, focus and depth range. To avoid operator influence, the US images were recorded without any operator intervention and the TGC was left set by the equipment. Three modes of images based on the frequency band were taken: “Resolution”, high frequency; “Penetration”, low frequency and “Compound”, broad-bandwidth. The images used for defining the biopsy site were stored on a computerized archiving system (PACS) and exported in a DICOM format to a PC (Pentium IV) for further processing. Image analysis was done using a special software package written by the authors in the Matalb® programming environment.

Among the three sets of images stored, the images obtained with the lower frequencies, i.e., “Penetration” imaging mode, were the most informative, probably due to the deeper penetration, that allowed useful measurements as far as the diaphragm. Thus, only the “Penetration” images were chosen for further analysis.

Histopathological analysis and oil red O staining

Upon being harvested from animals, murine tissues were fixed in 4% paraformaldehyde (PFA), embedded in paraffin and sectioned. HE staining were performed following standard procedures. Pathological parameters, including necrosis, infiltration of lymphocytes and monocytes, vascular formulation and fibrosis were evaluated.

For the oil red O (ORO) staining, liver tissues were fixed in 4% PFA and equilibrated with 30% sucrose, following with optimal cutting temperature (OCT) compound embedding, snap- freezing and sectioning. ORO staining was performed on cryosections using Lipid (Oil Red O) Staining Kit (Sigma, MAK194). The adipocyte size and numbers were measured using ImageScope-v12.0.1 software.

Lipidomic analysis

For the purpose of lipidomic analysis, liver and brain of mice were harvested and immediately stored in liquid nitrogen until extraction. Plasma were acquired from freshly collected blood by centrifugation and immediately frozen. Lipid extraction was performed following the standard procedure of chloroform-methanol method. The gas chromatography-mass spectrum was performed on the fatty acid extracts according to instructions of the manufacturer. Briefly, a standard curve was built with SPLASH® Lipidomix® Mass Spec Standard (SPLASH, 330707). Levels of saturated fatty acids, monounsaturated fatty acids and poly-unsaturated fatty acids were evaluated.

Glucose tolerance test and Insulin tolerance test

During GTT, an intraperitoneal injection of glucose (Sigma, G7528) with a single dose of 2 g/kg body weight was performed in 6h‐fasted mice. Blood samples were collected from the tail vein before glucose injection (0 min) and at 15, 30, 60 and 120 min afterward. Blood glucose concentration were immediately measured by a glucose meter (ONETOUCH Ultra, Lifescan).

ITT was performed by intraperitoneal injection of insulin (0.75 IU/Kg, Aladdin, 12584‐58‐6). Blood glucose concentrations were measured before insulin injection (0 min) and 30, 60, 90 and 120 min after insulin injection. Blood samples were collected from mice tail vain and blood glucose concentration were immediately measured by a glucose meter (ONETOUCH Ultra, Lifescan).

RNA-seq

Liver tissue and human RPE cell samples were collected and send to BGI company to perform RNA-seq. Total RNA was extracted from cultured cells (1 × 10^6^ cells) by TRIzol reagent (Invitrogen). RNA-Seq sequencing was performed on an Illumina HiSeq 4000 sequencer with 150 bp paired- end sequencing reactions.

The RNA-seq reads of each sample was mapped to the mouse mm9 or human hg19 genome assembly independently by the HISAT2 software ^13^ using the annotated gene structures as templates. Default parameters of HISAT2 were used except with the option “--dta-cufflinks” opening. Reads with unique genome location were reserved for gene expression calculation using Cufflinks (version 2.0.2) with the option “--GTF” ^14^. The heatmaps were produced by the heatmap.2 function of R. Gene ontology analysis of differentially expressed genes(The differentially expressed genes were identified by Cuffdiff with p value <= 0.0.5.) were analyzed in DAVID and processes were selected based on p values smaller than 0.05 ^15^. The differentially expressed gene list of mouse liver between aging and young were download from the published data ^16^. And the gene sets comparison between our data and the datasets were performed by Gene Set Enrichment Analysis (GSEA) software ^17^.

Immunofluorescence staining and MitoSOX staining

Murine tissue sample were freshly collected upon perfusion, followed by overnight PFA fixation and sucrose dehydration. Human fibroblast cells, human RPE cells and murine primary cells were fixed with 4% PFA for 1h. To perform immunofluorescence, the slide was rinsed in PBS for 5 minutes, blocked in blocking buffer (PBS with 1% BSA, 0.1% Tween-20) for 20 minutes at room temperature (RT), and incubated with primary antibody in blocking buffer for 1 hour at RT. After 3 washes with 0.1% Tween-20 in PBS, the slide was incubated with secondary antibody in blocking buffer for 1 hour at RT. The slides were mounted with DAPI-Vectashield solution (Vector laboratories). Images were taken with a confocal microscope (LSM 780). To estimate the mitochondrial conditions, human RPE cells and murine primary cells were incubated with either MitoSOX (5uM) according to manufacturer’s instructions.

Glycolysis stress test and Mito stress test

The glycolysis stress test and the mito stress test were performed on murine primary hepatocytes with Seahorse Bioscience XF Analyzer (Agilent Tech) following the instructions of manufacturer. Briefly, the murine hepatocytes were seed in the XF96 cell culture microplate (Seahorse Bioscience, 101085-004) with 100,000 cells per well. Ahead of the assays, the culture medium was replaced followed by 1h incubation in 37℃. For the glycolysis stress test, cell culture medium was replaced by Seahorse XF Base medium (Seahorse Bioscience), supplemented with L-glutamine. During the assay, glucose, oligomycin and 2-deoxyglucose were added into each well sequentially, followed by mixing and measurements. For the mito stress test, culture medium was replaced by Seahorse XF Base medium supplemented with glucose, l-glutamine and pyruvate. During the assay, oligomycin, FCCP and rotenone were added into each well sequentially, followed by mixing and measurements. Mixture time, incubation time and the timeline of chemicals addition were determined based according to instructions of manufacturer.

Magnetic resonance image acquisition

Axial, sagttial and coronal structure images of 8-week-old adult mice brain were acquired on a 7 Tesla MRI scanner (PharmScan 70/16 US, Bruker, Switzerland). The contrast required for registration and assessment of volume is not acceptable with our typical T2-weighted imaging sequence. Therefore, diffusion-weighted imaging was performed to enhance the contrast between white and grey matter to aid in the registration and volume measurements.

Quantification and statistical analysis

The annotation enrichment tests for CpG islands’ Methylation marker (Fig. 1a) were performed with the two-sided Fisher's exact test. Levels of significance for MeDip data (Fig. 1b, 1f) were calculated with one-tailed student’s t test. Levels of significance for BS analysis of DNA methylation in mouse tissues (Fig. 1c, S1c) were calculated with two tailed student’s t-test. Levels of significance for gene expression levels (Fig. 1e, S1e, S1h, 6d, S5c, S6a) were calculated with one-tailed student’s t test. Levels of significance for physical signs analysis (Fig. 3b, 3g, 3h, S2c, S3c, S5e) were calculated with two tailed student’s t-test. Levels of significance for pathological section analysis (Fig. 3d, 3f, 4c, 4d, 5d, S2g, S2h, S3d, S5d) were calculated with two tailed student’s t-test. Levels of significance for ELISA analysis (Fig. 4a, S4a, S4b, were calculated with two tailed student’s t-test. Levels of significance for Seahorse XF analysis (Fig. 5f, 5g) were calculated with one-tailed student’s t test. Levels of significance for cell doubling time analysis (Fig. 6b, S1g) were calculated with two tailed student’s t-test. Levels of significance for behavioristics analysis (Fig. 3c, S2d-f) were calculated with two tailed student’s t-test.

Reference

1 Steinegger, M. *et al.* HH-suite3 for fast remote homology detection and deep protein annotation. *BMC Bioinformatics* **20**, 473, doi:10.1186/s12859-019-3019-7 (2019).

2 Remmert, M., Biegert, A., Hauser, A. & Soding, J. HHblits: lightning-fast iterative protein sequence searching by HMM-HMM alignment. *Nat Methods* **9**, 173-175, doi:10.1038/nmeth.1818 (2011).

3 Buchan, D. W. A. & Jones, D. T. The PSIPRED Protein Analysis Workbench: 20 years on. *Nucleic Acids Res* **47**, W402-W407, doi:10.1093/nar/gkz297 (2019).

4 Jumper, J. *et al.* Highly accurate protein structure prediction with AlphaFold. *Nature* **596**, 583-589, doi:10.1038/s41586-021-03819-2 (2021).

5 Baek, M. *et al.* Accurate prediction of protein structures and interactions using a three-track neural network. *Science* **373**, 871-876, doi:10.1126/science.abj8754 (2021).

6 Fuchs, F., Worrall, D., Fischer, V. & Welling, M. SE(3)-Transformers: 3D Roto-translation equivariant attention networks. *Advances in Neural Information Processing Systems* **33**, 1970-1981 (2020).

7 Chaudhury, S., Lyskov, S. & Gray, J. J. PyRosetta: a script-based interface for implementing molecular modeling algorithms using Rosetta. *Bioinformatics* **26**, 689-691, doi:10.1093/bioinformatics/btq007 (2010).

8 Mariani, V., Biasini, M., Barbato, A. & Schwede, T. lDDT: a local superposition-free score for comparing protein structures and models using distance difference tests. *Bioinformatics* **29**, 2722-2728, doi:10.1093/bioinformatics/btt473 (2013).

9 Krammer, A., Kirchhoff, P. D., Jiang, X., Venkatachalam, C. M. & Waldman, M. LigScore: a novel scoring function for predicting binding affinities. *J Mol Graph Model* **23**, 395-407, doi:10.1016/j.jmgm.2004.11.007 (2005).

10 Gehlhaar, D. K. *et al.* Molecular recognition of the inhibitor AG-1343 by HIV-1 protease: conformationally flexible docking by evolutionary programming. *Chem Biol* **2**, 317-324, doi:10.1016/1074-5521(95)90050-0 (1995).

11 Muegge, I. & Martin, Y. C. A general and fast scoring function for protein-ligand interactions: a simplified potential approach. *J Med Chem* **42**, 791-804, doi:10.1021/jm980536j (1999).

12 Huda, N. *et al.* Recruitment of TRF2 to laser-induced DNA damage sites. *Free radical biology & medicine* **53**, 1192-1197, doi:10.1016/j.freeradbiomed.2012.07.024 (2012).

13 Kim, D., Langmead, B. & Salzberg, S. L. HISAT: a fast spliced aligner with low memory requirements. *Nature Methods* **12**, 357-360, doi:10.1038/nmeth.3317 (2015).

14 Trapnell, C. *et al.* Transcript assembly and quantification by RNA-Seq reveals unannotated transcripts and isoform switching during cell differentiation. *Nat Biotechnol* **28**, 511-515, doi:10.1038/nbt.1621 (2010).

15 Huang, D. W., Sherman, B. T. & Lempicki, R. A. Systematic and integrative analysis of large gene lists using DAVID bioinformatics resources. *Nat Protoc* **4**, 44-57, doi:10.1038/nprot.2008.211 (2009).

16 White, R. R. *et al.* Comprehensive transcriptional landscape of aging mouse liver. *BMC Genomics* **16**, 899, doi:10.1186/s12864-015-2061-8 (2015).

17 Subramanian, A. *et al.* Gene set enrichment analysis: a knowledge-based approach for interpreting genome-wide expression profiles. *Proc Natl Acad Sci U S A* **102**, 15545-15550, doi:10.1073/pnas.0506580102 (2005).


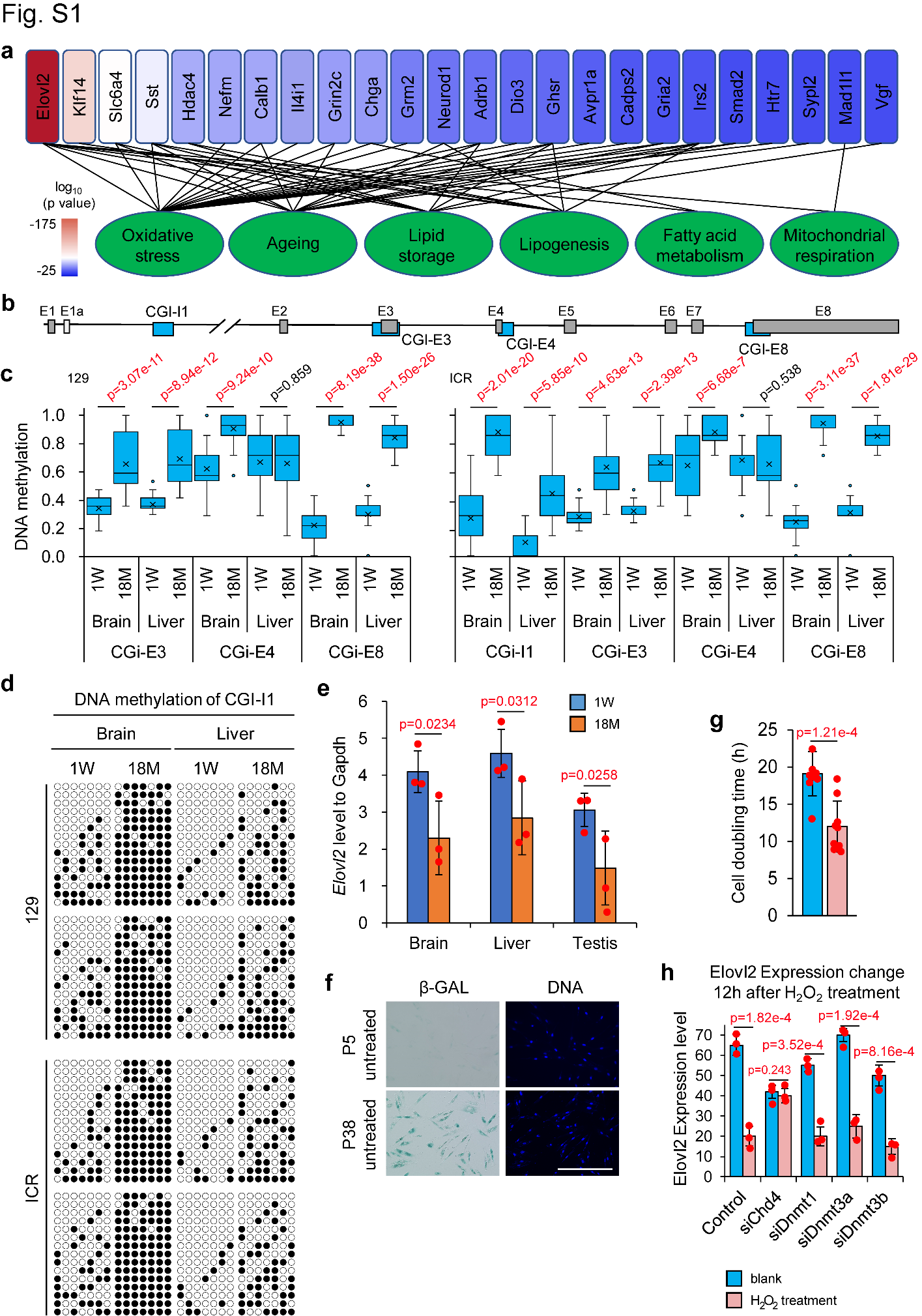


**Fig. S1 Elovl2 is a metabolic gene that serves as a strong marker of aging**. **(a)** Gene function of top Epigenetic aging markers. **(b)** The structure of Elovl2 in mouse. **(c)** The DNA methylation levels on the CpG islands of intron 1 (I1) and exon 3, 4, and 8 (E3, 4, 8) of Elovl2 in brain and liver of 129/sv and ICR mice. For each group 30 Sanger sequencing results from 6 mice were used. Levels of significance were calculated with two tailed student’s t-test. **(d)** The DNA methylation level on CGi-I1 in brain and liver of mice at the age of 18 months was significantly higher than that of 1 week. **(e)** *Elovl2* expression level decrease along with age (n = 3). Levels of significance were calculated with one tailed student’s t-test. Error bars, standard error of the mean (SEM). **(f)** Beta-galactosidase (β-GAL) staining on young (p5) and old (p38) human fibroblast cells (n = 3). Scale bar = 100 μm. **(g)** Representative statistical chart of cell proliferation (cell number doubling time) of human fibroblast cells with or without (blank) hydrogen peroxide (H_2_O_2_) treatment (n = 9). Levels of significance were calculated with two tailed student’s t-test. Error bars, SEM. **(h)** *Elovl2* expression level significantly decreased after H_2_O_2_ treatment in groups where DNMT1, DNMT3A or DNMT3B but not CHD4 were knockdown (n = 3). Levels of significance were calculated with one tailed student’s t-test.


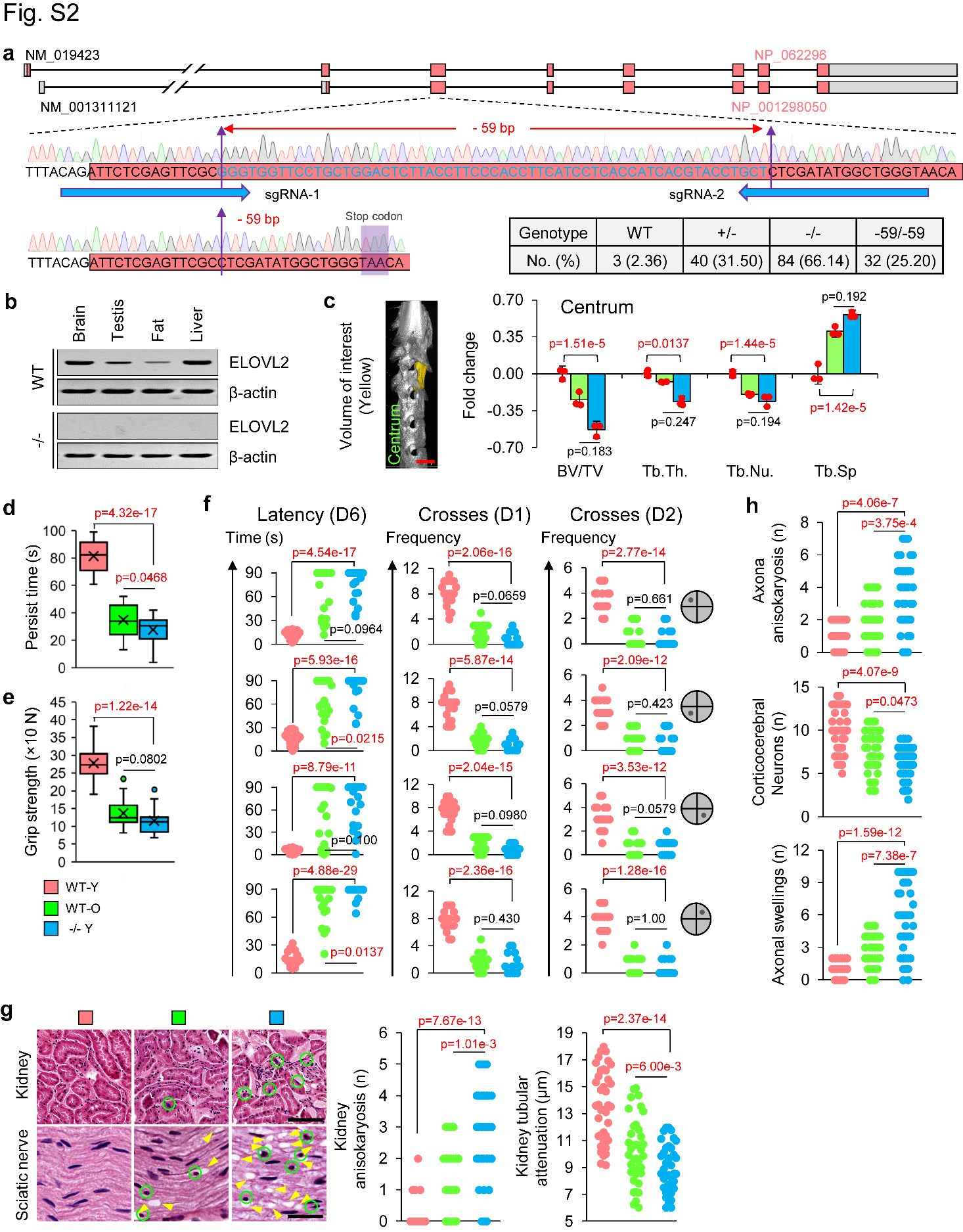


**Fig. S2 Deletion of Elovl2 causes dramatic acceleration of aging in mice. (a)** The design of sgRNA and genotype of mice in different groups. **(b)** Western blotting showing the expression of ELOVL2 in different organs of WT or -/- mice (n = 3). (**c**) Representative images showing the hair loss of WT-O or -/- mice. The bone volume/total volume (BV/TV), trabecular thickness (Tb. Th.), trabecular number (Tb. Nu.) and trabecular spacing (Tb. Sp.) was measured by Micro-CT. Error bars, SEM. Levels of significance were calculated with two tailed student’s t-test. Scale bar = 2 mm. (**d & e**) -/- Y mice displayed reductions in endurance (**d**) and muscle strength (**e**) (n = 20 per group). Error bars, SEM. Levels of significance were calculated with two tailed student’s t-test. (**f**) The Morris Water Maze test showed spatial learning and memory of WT-O and -/- mice were significantly decreased (n = 20 per group). Error bars, SEM. Levels of significance were calculated with two tailed student’s t-test. **(g & h**) Representative images of hematoxylin and eosin staining and pathological section analysis of kidney and sciatic nerve; green cycles show the abnormal structures. Scale bar = 100 µm. For each group 40 slices from 5 mice were sed for statistical analysis. Levels of significance were calculated with two tailed student’s t- test.


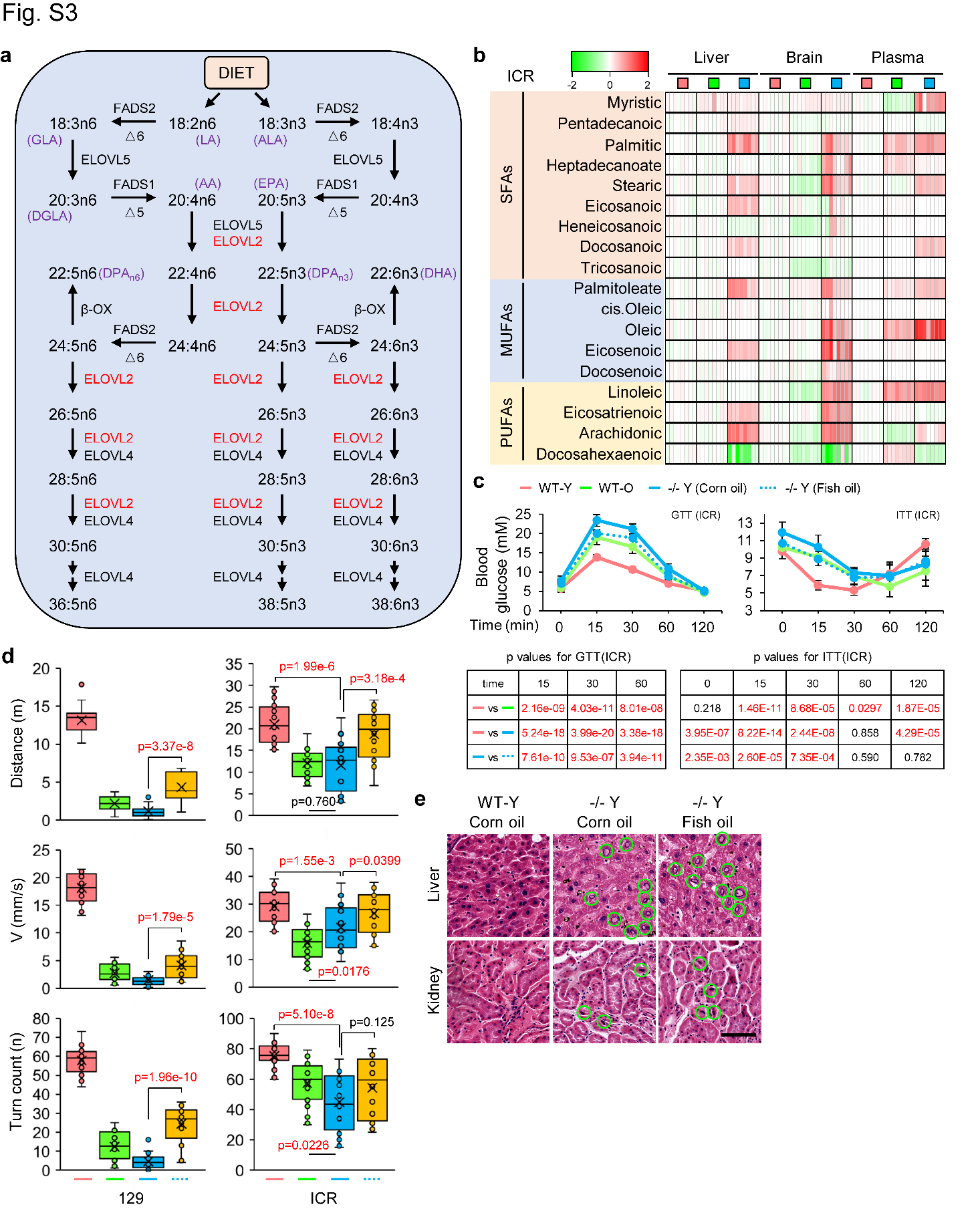


**Fig.S3 Multiple metabolic disturbances were found in Elovl2 KO mice. (a)** The schematics showing the roles of ELOVL families in lipid metabolism. **(b)** The heat map of fatty acid species, saturated fatty acids (SFAs), mono-unsaturated fatty acids (MUFAs) and poly-unsaturated fatty acids (PUFAs) in liver, brain and plasma of ICR mice (n = 8 per group, fold change in log2 scaled is used). **(c)** Glucose tolerance test (GTT) (left) and Insulin tolerance test (ITT) (right) for mice on regular diet (Corn oil) and PUFAs adding diet (Fish oil) (n = 12 per group). Error bars, SEM. Levels of significance were calculated with two tailed student’s t-test. **(d)** Distance, velocity and turn count of different groups of 129/sv mice and ICR mice fed with either corn oil or fish oil in the open-field test (n = 20 per group). Levels of significance were calculated with two tailed student’s t-test. **(e)** Representative images of hematoxylin and eosin staining and pathological section analysis of liver and kidney from WT-Y, -/- Y fed with corn oil supplemented diet and -/- Y fed with fish oil supplemented diet. Scale bar = 100 µm.


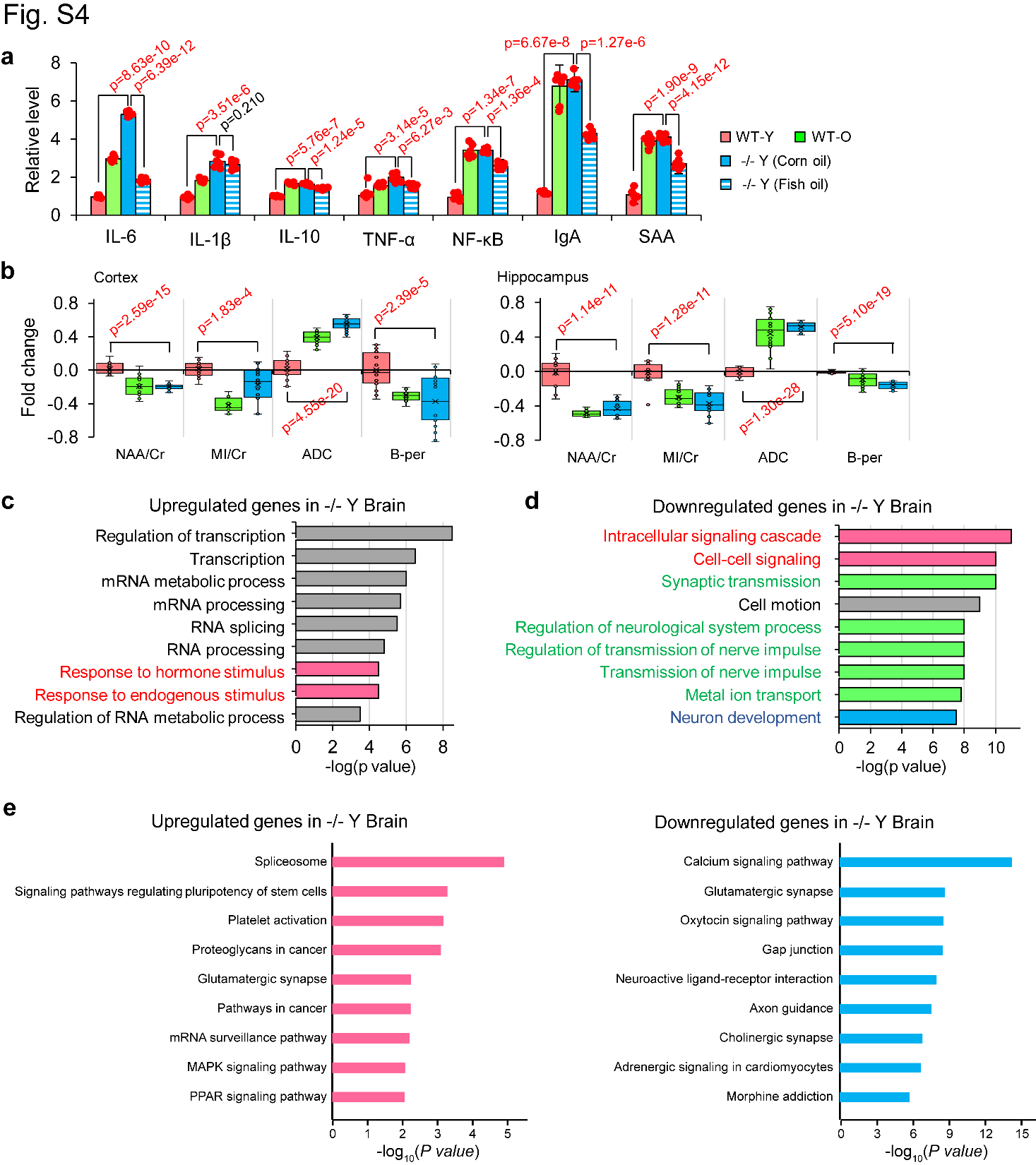


**Fig. S4 The depletion of Elovl2 in mice led to chronic inflammation and a decline in the function of eye and brain. (a)** ELISA test on inflammatory factors in blood samples from different groups of ICR mice (n = 6). Error bars, SEM. Levels of significance were calculated with two tailed student’s t-test. **(b)** Magnetic resonance imaging (MRI) analysis on the cerebral cortex and hippocampus (n = 20 per group). For each indicator, fold Changes to WT-Y group are used. It revealed a dramatic abnormity in -/- Y and WT-O mice. Levels of significance were calculated with two tailed student’s t-test. (**c & d**) Gene Ontology analysis of the RNA-Seq data revealed disfunction in the brain of -/-Y mice (n = 3 per group). The upregulated genes and downregulated genes are different expression genes between WT-Y and -/- Y mice analyzed by Cuffdiff with p value <= 0.0.5. **(e)** KEGG analysis for the DEGs genes in the brain samples, the enriched pathways are consistent with the Gene ontology results in general. The differentially expressed genes were identified by Cuffdiff with p value <= 0.0.5.


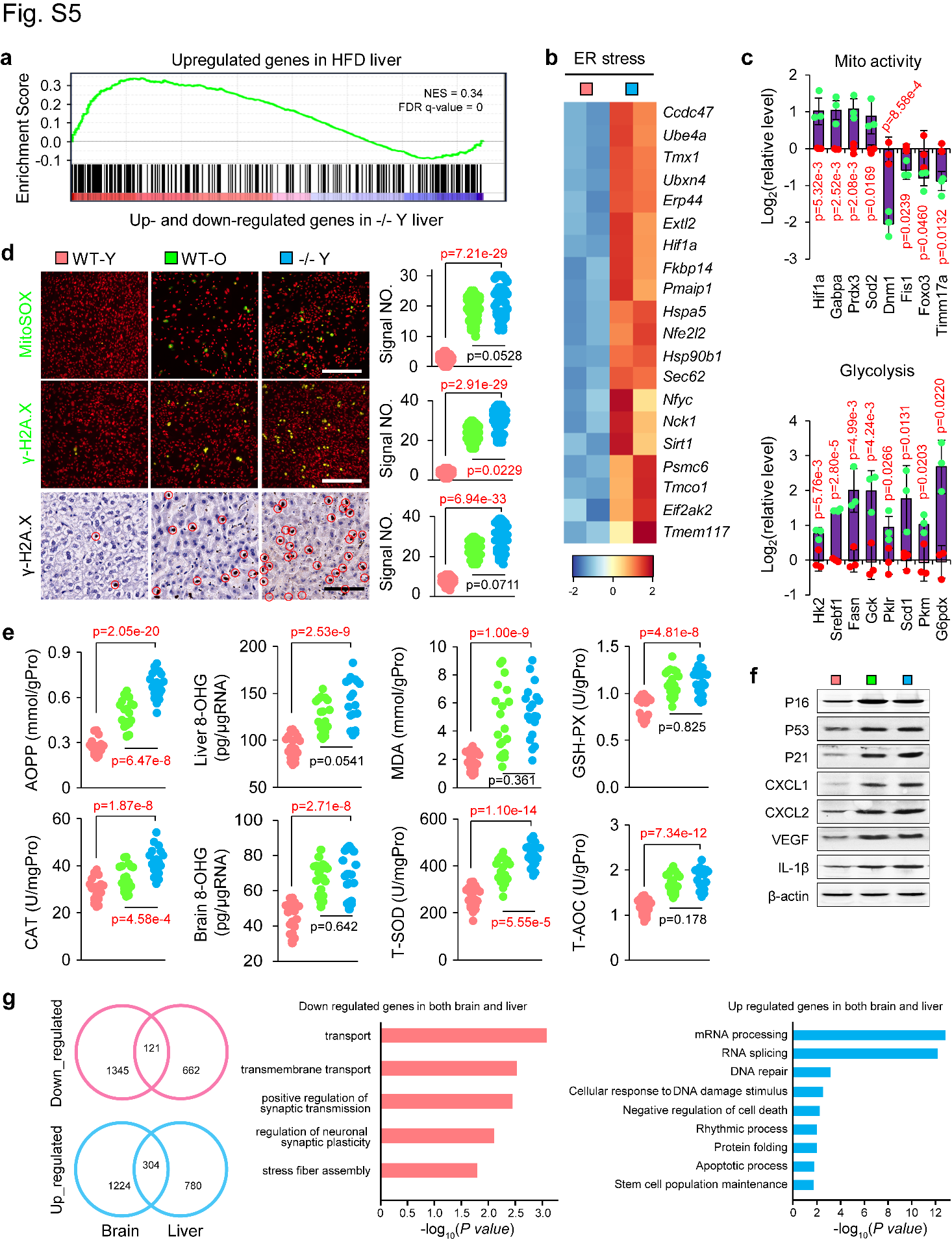


**Fig. S5 Elovl2 ablation leads to severe oxidative damage at the cellular level. (a)** Enriched gene sets of differentially expressed genes in -/- Y samples. The horizontal axis represents the differentially expressed genes in -/- Y compared to high fat diet (HFD) mouse samples which were ranked as either up- or down-regulated in -/- Y and marked in red and blue, respectively. The normalized enrichment score (NES) and false discovery rate (FDR) are marked. **(b)** Expression pattern of genes in ER stress pathways in WT-Y and -/- Y liver samples. **(c)** qPCR results verified the RNA-Seq data (n = 2). Error bars, SEM. Levels of significance were calculated with one tailed student’s t-test. **(d)** MitoSOX staining and γH2A.X staining showed sever oxidative damage in mitochondria and nuclei respectively in -/- Y mice. Error bars, SEM. Levels of significance were calculated with two tailed student’s t-test. For each group 40 slices from 5 mice were sed for statistical analysis. Scale bar = 100 µm. **(e)** The oxidative damage affecting proteins (AOPP), lipids (MDA), and RNA (8-OHG) (n = 20 per group). Error bars, SEM. **, p <0.01. Levels of significance were calculated with two tailed student’s t-test. **(f)** Higher cellular senescent markers were detected by western blotting in -/- Y and WT-O mice (n = 3). **(g)** Analysis of differentially expressed overlap genes in both brain and liver of ELOVL2 -/- mouse. 121 overlap genes are down regulated and 304 over lap genes are upregulated.


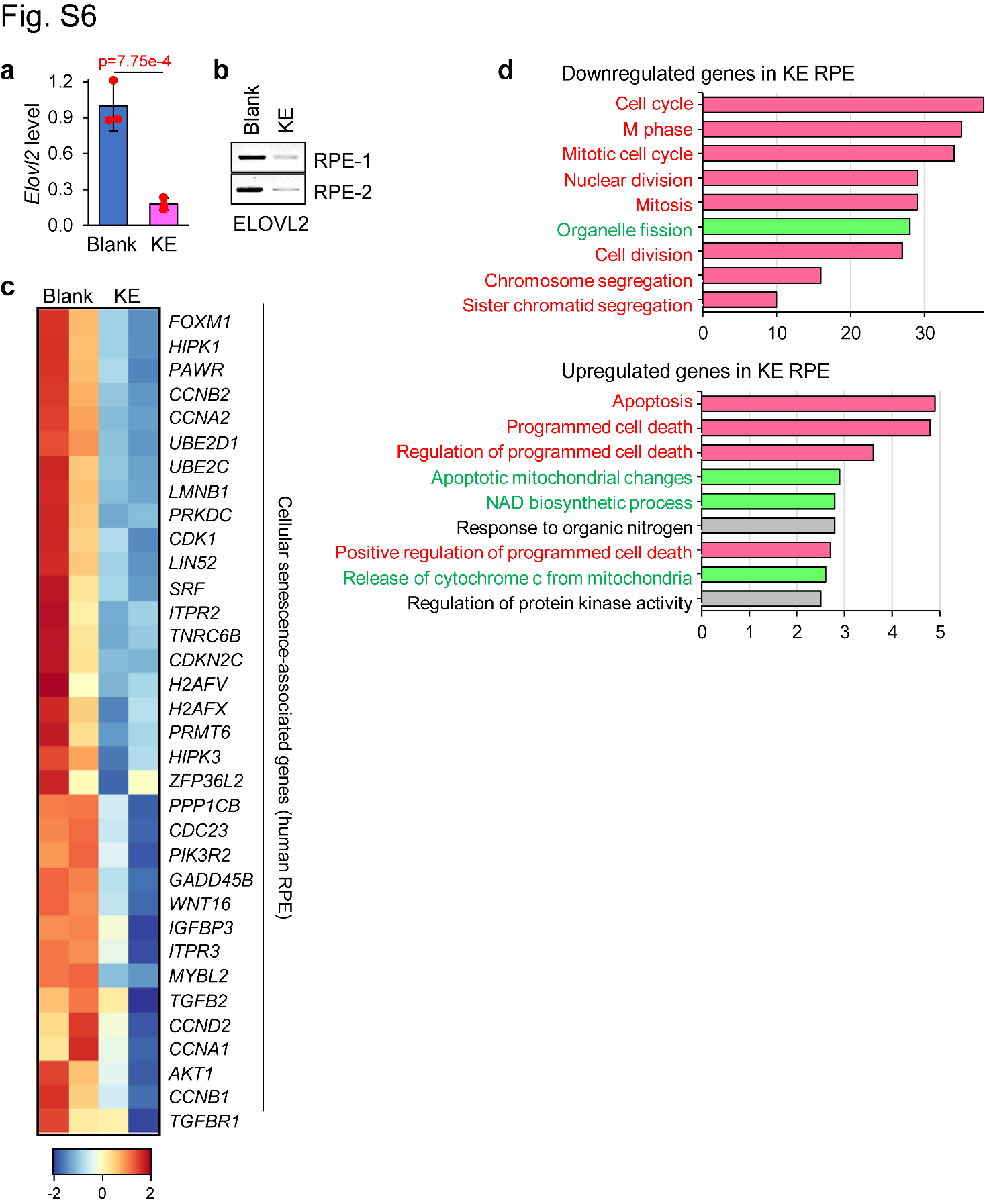


**Fig. S6 AMD phenotype was induced by *ELOVL2* deficiency. (a & b)** qPCR **(a)** and Western blotting **(b)** results showed ELOVL2 expressions in blank and KE human RPE cells (n = 3). Levels of significance were calculated with one tailed student’s t-test. **(c)** Heatmap showing the cluster of cellular senescence-associated genes with variation between blank and KE human RPE cells. **(d)** RNA-seq data revealed the upregulated genes and downregulated genes in KE human RPE cells.


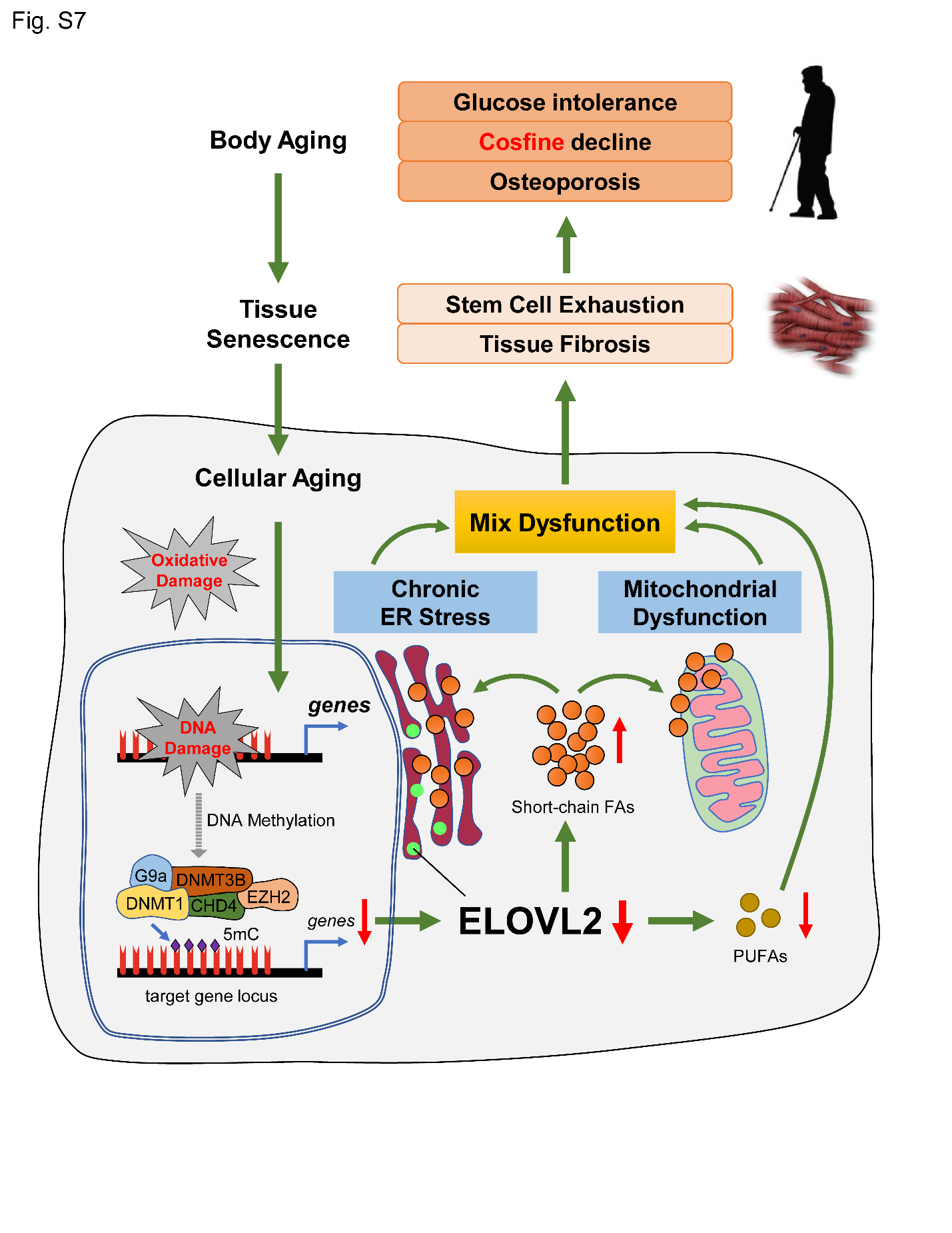


**Fig. S7 Schematic of model for age-related DNA methylation mediated accelerated aging process**


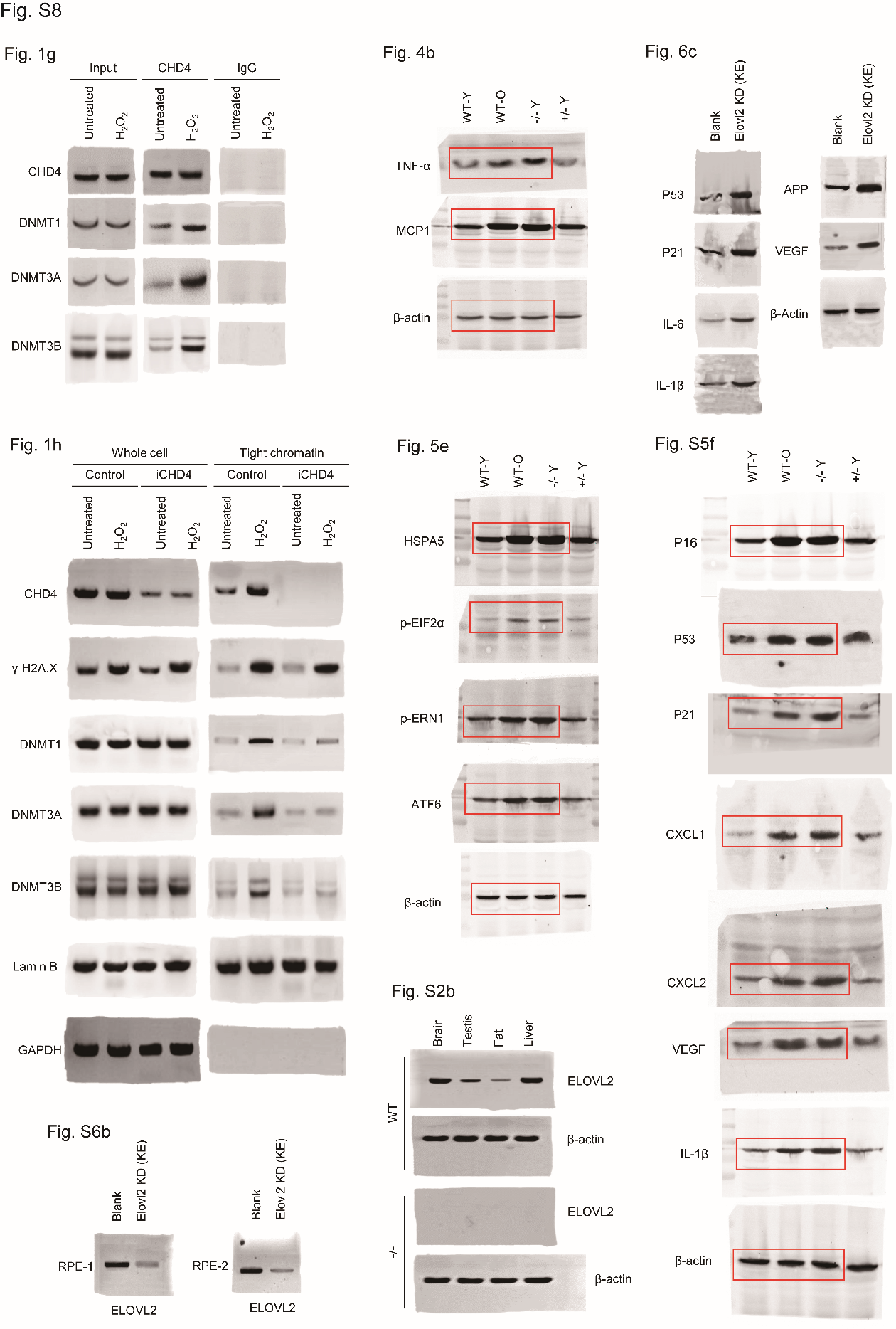


**Fig. S8 Original images for western blot.**

**Table S1. Primers used in this paper.**

| Used in | Name | Sequence |
| --- | --- | --- |
| MeDip-qPCR | hElovl2-MeDip-F | CTAATGTGTGCTTCAAACCCACCG |
|  | hElovl2-MeDip-R | GCGGCGATTTGCAGGTCCAG |
| BS-PCR | mElovl2-BSI1-F1 | AAAAAGAAATGTTTTTTATTATAAGTTA |
|  | mElovl2-BSI1-R1 | AACTATTTCTTCTACCTATCACATCTAC |
|  | mElovl2-BSI1-F2 | AAGAAATGTTTTTTATTATAAGTTATTTT |
|  | mElovl2-BSI1-R2 | TCTTCTACCTATCACATCTACTATAATATC |
|  | mElovl2-BSE3-F1 | TTTAATGTTGAGGTTGTTGAGAAAG |
|  | mElovl2-BSE3-R1 | CACAACTCTAAAAAATCCACCTAAC |
|  | mElovl2-BSE3-F2 | GTTGAGGTTGTTGAGAAAGAATTAG |
|  | mElovl2-BSE3-R2 | CAAAATACAAACCACAACCACTTAC |
|  | mElovl2-BSE4-F1 | AGTTGGGAAGGAGGTTATAATTTG |
|  | mElovl2-BSE4-R1 | CAATAATAATAATTCTAAAACTATCTATTCTC |
|  | mElovl2-BSE4-F2 | GGAGGTTATAATTTGTAGTGTTAGAAT |
|  | mElovl2-BSE4-R2 | AATAATAATTCTAAAACTATCTATTCTCAATA |
|  | mElovl2-BSE8-F1 | GTTTATTTAATTGTGGTTAATGGTATG |
|  | mElovl2-BSE8-R1 | TTACACTACAATAAACAATTTTACACATC |
|  | mElovl2-BSE8-F2 | ATAAGAAGGTTTAATAAAATGAAGTGT |
|  | mElovl2-BSE8-R2 | AACTCTAAAAACTTACCATAAAAAAAC |
| qPCR | hApoe-qF | GTTGCTGGTCACATTCCTGG |
|  | hApoe-qR | GCAGGTAATCCCAAAAGCGAC |
|  | hApoj-qF | CTACTTCTGGATGAATGGTGACC |
|  | hApoj-qR | CGGGTGAAGAACCTGTCCT |
|  | hApp-qF | TCTCGTTCCTGACAAGTGCAA |
|  | hApp-qR | GCAAGTTGGTACTCTTCTCACTG |
|  | hClpp-qF | GCTCAAGAAGCAGCTCTATAACATCTACG |
|  | hClpp-qR | GGTGGACCAGAACCTTGTCTAAGATG |
|  | hCryab-qF | AGGTGTTGGGAGATGTGATTGA |
|  | hCryab-qR | GGATGAAGTAATGGTGAGAGGGT |
|  | hCxcl1-qF | GCTCACTGGTGGCTGTTCCTG |
|  | hCxcl1-qR | CTCAAACACATTAGGCACAATCCAG |
|  | hDnm1-qF | GACATCGAGTTCCAGATCCGAGAC |
|  | hDnm1-qR | CATCAGGTCCAGCTTGGTGATGA |
|  | hElovl2-qF | CCAAATCAGTAGAGTTCCTGGACACAA |
|  | hElovl2-qR | TTGTCCACAAGGTATCCAGTTCAAGAC |
|  | hFis1-qF | ACAGCGGGATTACGTCTTCTACCTG |
|  | hFis1-qR | AGGATTTGGACTTGGACACAGCAA |
|  | hGapdh-qF | CATCCTGGGCTACACTGAGCACC |
|  | hGapdh-qR | AAGTGGTCGTTGAGGGCAATGC |
|  | hIl13-qF | AGAGGATGCTGAGCGGATTCTG |
|  | hIl13-qR | AGAGCAGGTCCTTTACAAACTGGG |
|  | hIl1b-qF | ATGATGGCTTATTACAGTGGCAATG |
|  | hIl1b-qR | CGGAGATTCGTAGCTGGATGC |
|  | hIl6-qF | GCCACTCACCTCTTCAGAACGAA |
|  | hIl6-qR | CCATCTTTGGAAGGTTCAGGTTG |
|  | hIl8-qF | ACTGAGAGTGATTGAGAGTGGAC |
|  | hIl8-qR | AACCCTCTGCACCCAGTTTTC |
|  | hMcp1-qF | CAGCCAGATGCAATCAATGCC |
|  | hMcp1-qR | TGGAATCCTGAACCCACTTCT |
|  | hNmnat1-qF | GGTGGAAGTTGATACATGGGAAAGTC |
|  | hNmnat1-qR | GAGTAGGTGAGTTCTGCTGGTGATCAC |
|  | hNmnat2-qF | CCATTTACCAGAACAGCAACGTGG |
|  | hNmnat2-qR | GTGCCCAGATTGGCATTCTCATC |
|  | hOpn-qF | GAAGTTTCGCAGACCTGACAT |
|  | hOpn-qR | GTATGCACCATTCAACTCCTCG |
|  | hPgc1a-qF | TCTGAGTCTGTATGGAGTGACAT |
|  | hPgc1a-qR | CCAAGTCGTTCACATCTAGTTCA |
|  | hPgc1b-qF | GATGCCAGCGACTTTGACTC |
|  | hPgc1b-qR | ACCCACGTCATCTTCAGGGA |
|  | hPhb2-qF | GGGCTGGACTACGAGGAACGAG |
|  | hPhb2-qR | GCTGTGAGGCATTGAACTTGGC |
|  | hPmpcb-qF | TTGAACGTGAGCGTGGAGTAATCC |
|  | hPmpcb-qR | GTGCAGTATTTTGATAAGCTGTGGCA |
|  | hTxn2-qF | TTCAAGACCGAGTGGTCAACAGTG |
|  | hTxn2-qR | TGACACCTCATACTCAATGGCGAG |
|  | hVegfa-qF | GAGGGCAGAATCATCACGAAGTGG |
|  | hVegfa-qR | TGGAAGATGTCCACCAGGGTCTCGA |
|  | hVtn-qF | TGACCAAGAGTCATGCAAGGG |
|  | hVtn-qR | ACTCAGCCGTATAGTCTGTGC |
|  | mAgt-qF | TCTCCTTTACCACAACAAGAGCA |
|  | mAgt-qR | CTTCTCATTCACAGGGGAGGT |
|  | mAnt2-qF | ATAGACTGCGTGGTTCGTATCC |
|  | mAnt2-qR | GGGGAAGTATCTGATGACATTGG |
|  | mAnxa2-qF | ATGTCTACTGTCCACGAAATCCT |
|  | mAnxa2-qR | CGAAGTTGGTGTAGGGTTTGACT |
|  | mApp-qF | TCCGAGAGGTGTGCTCTGAA |
|  | mApp-qR | CCACATCCGCCGTAAAAGAATG |
|  | mAtf6-qF | CAAGACTCAAACCAATGCCAGTGTC |
|  | mAtf6-qR | CTGTATGCTGATAATCGACTGCTGCT |
|  | mCanx-qF | ATAAAGGACTTGTACTGATGTCTCGGG |
|  | mCanx-qR | CTTCACATAGGCACCACCACATTCTA |
|  | mCox5b-qF | GGAAGACCCTAATCTAGTCCCG |
|  | mCox5b-qR | GTTGGGGCATCGCTGACTC |
|  | mCxcl1-qF | ACTGCACCCAAACCGAAGTC |
|  | mCxcl1-qR | TGGGGACACCTTTTAGCATCTT |
|  | mCxcl2-qF | CCAACCACCAGGCTACAGG |
|  | mCxcl2-qR | GCGTCACACTCAAGCTCTG |
|  | mDnaja1-qF | GTGAAGGCCGAGGTGGTAAG |
|  | mDnaja1-qR | TGCACACTGACTGAATTTGCT |
|  | mDnm1-qF | AGAATATGCCGAGTTCCTGCACTG |
|  | mDnm1-qR | CGGTCTCAGCCTCGATCTCCA |
|  | mEif2a-qF | CACACCGCTGTTGACAGTCAGAG |
|  | mEif2a-qR | GGCAAACAATGTCCCATCCTTACTA |
|  | mElovl2-qF | TCACGTACCTGCTCTCGATATGGC |
|  | mElovl2-qR | GATGAGCTCCACCAGCATATACGC |
|  | mErn1-qF | ACCGACCACCGTATCTCAGGATG |
|  | mErn1-qR | TGCTCAGGATAATGGTAGCCATGTC |
|  | mFasn-qF | CAGAGATCCCGAGACGCTTCTG |
|  | mFasn-qR | AATGCTTGGTCCTTTGAAGTCGAA |
|  | mFis1-qF | AAAGGCTCTAAAGTATGTGCGAGGG |
|  | mFis1-qR | ATGGCCTTATCAATCAGGCGTTC |
|  | mFoxo3-qF | CTTCATGCGCGTTCAGAATGAAG |
|  | mFoxo3-qR | GAAGGACTGTCGTCTGCCGACTC |
|  | mG6pdx-qF | CACAGTGGACGACATCCGAAA |
|  | mG6pdx-qR | AGCTACATAGGAATTACGGGCAA |
|  | mGabpa-qF | AGCGCATCTCGTTGAAGAAG |
|  | mGabpa-qR | TCCTGCTCTTTTCTGTAGCCT |
|  | mGapdh-qF | AAGGCTGTGGGCAAGGTCATC |
|  | mGapdh-qR | GTCATCATACTTGGCAGGTTTCTCC |
|  | mGck-qF | AAGACGAAACACCAGATGTATTCCATC |
|  | mGck-qR | AGCCCTTGGTCCAGTTGAGCA |
|  | mHif1a-qF | GGGGAGGACGATGAACATCAA |
|  | mHif1a-qR | GGGTGGTTTCTTGTACCCACA |
|  | mHk2-qF | ATGATCGCCTGCTTATTCACG |
|  | mHk2-qR | CGCCTAGAAATCTCCAGAAGGG |
|  | mHsp90b1-qF | GCACTCGCTGGAAATGAGGAGTTA |
|  | mHsp90b1-qR | GAGGTTGACTGACCATCTTCTTGAGC |
|  | mHspa5-qF | CGTACATTCAAGTTGATATTGGAGGTG |
|  | mHspa5-qR | AGCATCTTTGGTTGCTTGTCGC |
|  | mIl1b-qF | GCAACTGTTCCTGAACTCAACTGTG |
|  | mIl1b-qR | AGCTGGATGCTCTCATCAGGACA |
|  | mMcp1-qF | CATTAAAAACCTGGATCGGAACCA |
|  | mMcp1-qR | GCATTAGCTTCAGATTTACGGGTCA |
|  | mNfe2l2-qF | TCTTGGAGTAAGTCGAGAAGTGT |
|  | mNfe2l2-qR | GTTGAAACTGAGCGAAAAAGGC |
|  | mP16-qF | GGACATCAAGACATCGTGCGATAT |
|  | mP16-qR | CGTGCTTGAGCTGAAGCTATGC |
|  | mP21-qF | AATCCTGGTGATGTCCGACCTG |
|  | mP21-qR | CCATGAGCGCATCGCAATC |
|  | mP53-qF | GTCACAGCACATGACGGAGGTC |
|  | mP53-qR | TGTCTTCCAGATACTCGGGATACAA |
|  | mPdia3-qF | TGATGGAATTGTCAGCCACTTGAAG |
|  | mPdia3-qR | GTTGGTGTGTGCAAATCGGTAGTTATC |
|  | mPklr-qF | CTCATCTCCTTAGTGGTGCGGAAA |
|  | mPklr-qR | TCGGCATTTGGCAAGTTCACAC |
|  | mPkm-qF | GGTGGCTCTGGATACAAAGGG |
|  | mPkm-qR | ACTTCTCCATGTAAGCGTTGTC |
|  | mPrdx3-qF | GGTTGCTCGTCATGCAAGTG |
|  | mPrdx3-qR | CCACAGTATGTCTGTCAAACAGG |
|  | mPsmc6-qF | ATGGCGGACCCTAGAGATAAG |
|  | mPsmc6-qR | TCTGTCCAACACTTTGTAGTGC |
|  | mScd1-qF | TCTTGCGATACACTCTGGTGCTCA |
|  | mScd1-qR | GGGATTGAATGTTCTTGTCGTAGGG |
|  | mSod2-qF | CAGACCTGCCTTACGACTATGG |
|  | mSod2-qR | CTCGGTGGCGTTGAGATTGTT |
|  | mSpp1-qF | AGCAAGAAACTCTTCCAAGCAA |
|  | mSpp1-qR | GTGAGATTCGTCAGATTCATCCG |
|  | mSrebf1-qF | TGTAGGTCACCGTTTCTTTGTGGAC |
|  | mSrebf1-qR | GCTGGGCTGAGCAATACAGTTCA |
|  | mTimm17a-qF | TGCCCCTGGCGAATTGTAG |
|  | mTimm17a-qR | CTGTCAAACTTCCTCGGAGTC |
|  | mTmem33-qF | GGCTGCATCAGAGATTACCTC |
|  | mTmem33-qR | TGACAGGGTAGGAGTTGACGA |
|  | mTnf-qF | TACTGAACTTCGGGGTGATCGG |
|  | mTnf-qR | AGGCTTGTCACTCGAATTTTGAGAA |
|  | mUcp2-qF | GTGGTGGTCGGAGATACCAGAGC |
|  | mUcp2-qR | TCAGCACAGTTGACAATGGCATTAC |
|  | mVegfa-qF | GCACATAGAGAGAATGAGCTTCC |
|  | mVegfa-qR | CTCCGCTCTGAACAAGGCT |
| in vitro transcription of sgRNA | T7-sg1-F | TAATACGACTCACTATAGGGCAGATTCTC  GAGTTCGCGGGGTTTTAGAGCTAGAAATA  GCAAG |
|  | T7-sg2-F | TAATACGACTCACTATAGGGTACCCAGCC  ATATCGAGAGCGTTTTAGAGCTAGAAATA  GCAAG |
|  | sg-BB-R | AAGCACCGACTCGGTGCCACTTTTTC |

**Table S2. GO terms for DEGs in -/-Y liver**

|  | Term | -lg(p_value) | p_value |
| --- | --- | --- | --- |
| GO terms for downregulated genes in -/-Y liver | Transmembrane transport | 6.200680432 | 6.30E-07 |
|  | Response to hormone stimulus | 4.300577695 | 5.01E-05 |
|  | Lipid biosynthetic process | 4.100409253 | 7.94E-05 |
|  | Oxidation reduction | 4.000733634 | 9.98E-05 |
|  | Insulin receptor signaling pathway | 3.50074007 | 3.16E-04 |
|  | Phospholipid biosynthetic process | 3.000126432 | 1.00E-03 |
|  | Cellular response to insulin stimulus | 3.000931973 | 9.98E-04 |
|  | Cellular homeostasis | 2.900682879 | 1.26E-03 |
|  | Cellular response to hormonr stimulus | 2.800638844 | 1.58E-03 |
| GO terms for upregulated genes in -/-Y liver | mRNA metabolic process | 8.500005245 | 3.16E-09 |
|  | Protein folding | 6.000585879 | 9.99E-07 |
|  | Fatty acid metabolic process | 5.50088669 | 3.16E-06 |
|  | Oxidation reduction | 5.500700763 | 3.16E-06 |
|  | Transcription | 3.500712038 | 3.16E-04 |
|  | RNA splicing | 3.500656232 | 3.16E-04 |
|  | Protein complex assembly | 3.000986281 | 9.98E-04 |
|  | mRNA processing | 2.900750593 | 1.26E-03 |
|  | Regulation of transcription | 2.900941657 | 1.26E-03 |
